# Classification of the tongue microbiota and its associations with lifestyle factors and health status

**DOI:** 10.1038/s41522-026-00936-6

**Published:** 2026-02-26

**Authors:** Toshitaka Yamauchi, Naoko Waki, Shigenori Suzuki, Tsukasa Tanaka, Shintaro Yokoyama, Koichi Murashita, Tatsuya Mikami, Yoshinori Tamada, Ken Itoh, Yoshihiro Tamura, Wataru Kobayashi

**Affiliations:** 1 Department of Vegetable Life Science, Graduate School of Medicine, Hirosaki University, Hirosaki, Japan; 2 Department of Medical Data Intelligence, Research Center for Health-Medical Data Science, Graduate School of Medicine, Hirosaki University, Hirosaki, Japan; 3 Diet & Well-being Research Institute, KAGOME CO. LTD., Nasushiobara, Japan; 4 Innovation Center for Health Promotion, Hirosaki University Graduate School of Medicine, Hirosaki, Japan; 5 Research Institute of Health Innovation, Hirosaki University, Hirosaki, Japan; 6 Department of Preemptive Medicine, Innovation Center for Health Promotion, Hirosaki University Graduate School of Medicine, Hirosaki, Japan; 7 Department of Stress Response Science, Biomedical Research Center, Hirosaki University Graduate School of Medicine, Hirosaki, Japan; 8 Department of Oral and Maxillofacial Surgery, Hirosaki University Graduate School of Medicine, Hirosaki, Japan

**Keywords:** Computational biology and bioinformatics, Diseases, Health care, Medical research, Microbiology

## Abstract

The oral microbiota plays a vital role in human health, yet most studies have focused on individual bacterial taxa. To provide a more comprehensive understanding, we analyzed tongue microbiota data from 729 Japanese individuals and classified samples into three types (orotypes): *Neisseria*-dominant (N), *Prevotella*-dominant (P), and *Streptococcus*-dominant (S) types. Each orotype exhibited distinct co-occurrence network structures and was associated with lifestyle factors such as oral care, diet, and smoking. The S type was associated with higher odds of abnormal oral health and metabolic syndrome-related outcomes compared to the N type. In addition, we developed a robust classification model (ROC–AUC > 0.95) to predict orotypes, which showed temporal stability in nearly half of individuals over a 6-year period. These findings highlight the value of orotype classification for monitoring tongue microbial communities and suggest its potential in health risk assessment.

## Introduction

In humans, microbiomes coexist in various tissues, including the gut, oral cavity, and skin^1^. The oral cavity harbors the second most abundant bacterial community, which influences not only oral health, but also systemic health^2–5^. For example, oral bacteria have been associated with cardiovascular, neurodegenerative, and inflammatory conditions^2–5^. The proposed mechanism involves bacterial components, such as cell walls and secreted proteins, which trigger local inflammation, subsequently leading to systemic inflammation^2,6^. Therefore, the oral microbiome may influence a wide range of health conditions through systemic inflammation, and maintaining oral hygiene is essential for prevention.

Most studies on the relationship between oral microbiome and health status have focused on each bacterial taxon separately^7–9^. *Porphyromonas gingivalis*, *Treponema denticola*, and *Tannerella forsythia* are involved in periodontal disease^7^, whereas *Streptococcus mutans* is associated with dental caries^8^. Owing to limited physical space and nutrient availability, the oral microbiome is highly competitive^10,11^. At the same time, the oral microbiome also exhibits symbiotic interactions with different bacterial species compensating for each other’s metabolic shortcomings^12,13^. Therefore, evaluating the oral microbiome comprehensively is also important. However, few studies have investigated the relationship between the overall oral microbiome—particularly the tongue microbiota—rather than individual bacterial taxa, and health status^14–16^.

Comprehensive analyses of gut microbiome data have been performed in several studies^17–19^. The first analytical approach in this sense is enterotyping, which is a method of classifying individuals into several types based on the relative abundance of the overall bacterial genera^17^. Consequently, the gut microbiome is classified into three to five enterotypes, each associated with various diseases or biomarkers^17,18^. The second analytical approach is co-occurrence analysis, which is a method of visualizing competitive and symbiotic relationships^20^. This type of analysis has revealed that the interacting bacterial communities differ between healthy individuals and patients, and that the strength of their associations varies^19^. Taken together, comprehensive analyses have revealed unique findings that could not have been uncovered by approaches focusing on separate bacterial taxa, thereby contributing to the improvement of disease prevention and treatment strategies by targeting the overall microbiome.

The establishment of oral bacterial communities is influenced more by environmental factors than host genetic background^21^. Oral hygiene practices such as tooth brushing and professional mechanical tooth cleaning have a significant impact on the oral microbiome^22,23^, as do smoking, alcohol consumption, and dietary habits^24–32^. However, no comprehensive study has analyzed the association between the tongue microbiome and lifestyle factors or health status.

In this study, we aimed to classify the tongue microbiota types (orotypes) and to examine its associations with lifestyle factors and health status using data from the Iwaki Health Promotion Project (IHPP).

## Results

### Orotype classification

**Fig. 1:**
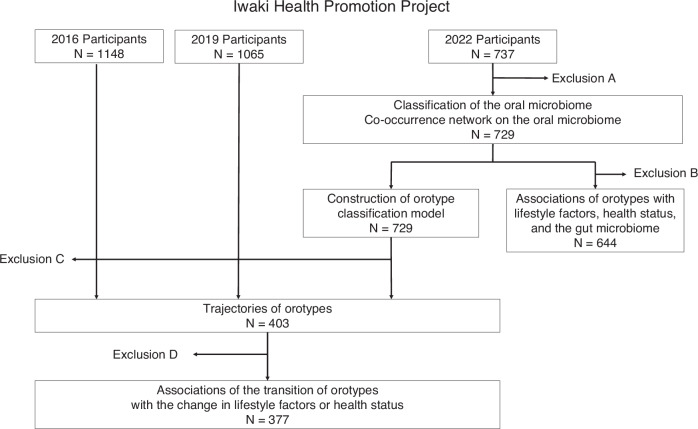
Flowchart of participant selection. Exclusion A: Missing data on tongue microbiota. Exclusion B: Missing data on lifestyle factors or health status in 2022. Exclusion C: Nonparticipation in any year or missing data on tongue microbiota. Exclusion D: Missing data on lifestyle factors or health status in any year.

This study included participants from the 2022 IHPP, an annual health checkup program for men and women aged ≥20 years living in northern Japan. Orotype classification was performed for 729 included participants, after individuals with missing tongue microbiota data were excluded (Fig. 1, Exclusion A).

**Fig. 2:**
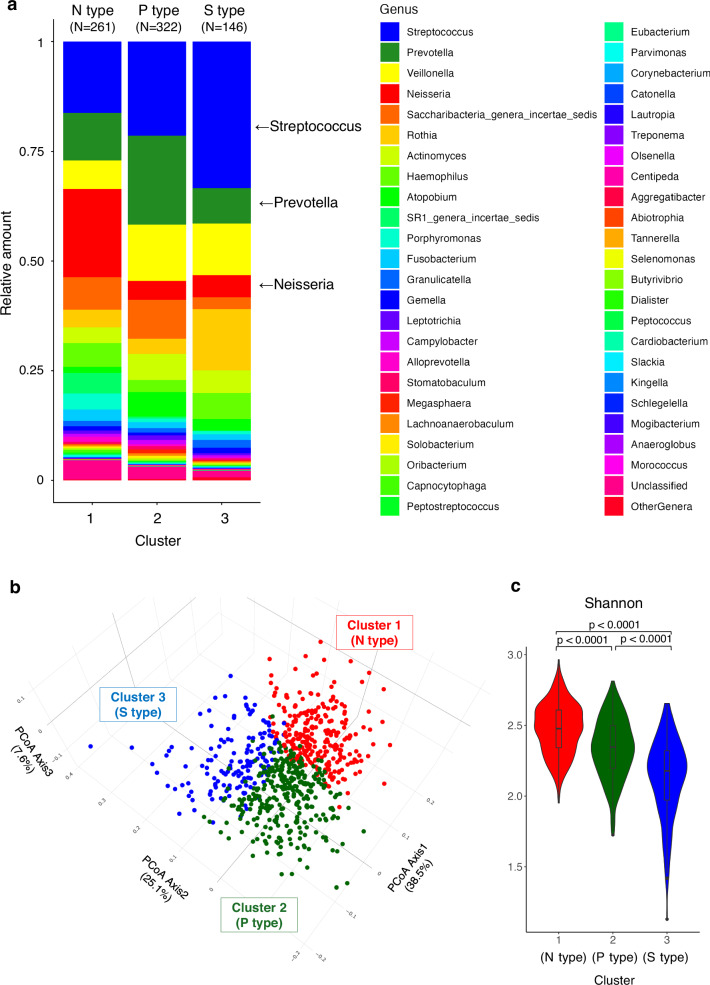
Classification of the tongue microbiota. **a** Stacked bar plots of the relative abundance of tongue bacterial genera by orotype. **b** Distribution of the tongue microbiota using Principal Coordinates Analysis (PCoA). Each plot color corresponds to an orotype. **c** Violin plot of α-diversity (Shannon index) by orotype. *P*-values were calculated using the Wilcoxon rank sum test with Bonferroni correction.

The number of orotypes was determined using the Calinski–Harabasz index (CH Index)^33^. Clustering into two or three groups provided the most distinct population separation (Supplementary Fig. 1a). Stacked bar plots illustrate the average composition of the tongue bacterial genera in each cluster for both the two- and three-cluster models (Fig. 2a, Supplementary Fig. 1b). In the three-cluster model, *Neisseria*, *Prevotella*, and *Streptococcus* were characteristically abundant in each orotype. Accordingly, they were designated as the N, P, and S types, respectively. The distribution of participants across the three types was as follows: N type (*n* = 261, 35.8%), P type (*n* = 322, 44.2%), and S type (*n* = 146, 20.0%). A transition diagram illustrates the changes in the orotype classification for each individual across the two-to four-cluster models (Supplementary Fig. 1c). In the two-cluster model, the population is divided into N and P types. By increasing the number of clusters to three, a minor proportion of individuals from each cluster was separated, creating the S type. However, classification in the four-cluster model differed substantially from that in the three-cluster model. To maximize the number of clusters while preserving clustering resolution, only the results from the three-cluster model are shown hereafter.

The distribution of individuals’ tongue microbiota by orotype was visualized using Principal Coordinate Analysis (Fig. 2b). The N type showed the highest α-diversity (Shannon index), followed by the P and then the S types (Fig. 2c). The relative abundance of each genus by orotype is shown in Supplementary Table 1. Moreover, in species-level analyses, we found that bacterial species belonging to representative genera (*Neisseria*, *Prevotella*, and *Streptococcus*) were generally abundant in their corresponding orotypes (Supplementary Fig. 2, Supplementary Table 1).

### Tongue microbiota co-occurrence network analysis

**Fig. 3:**
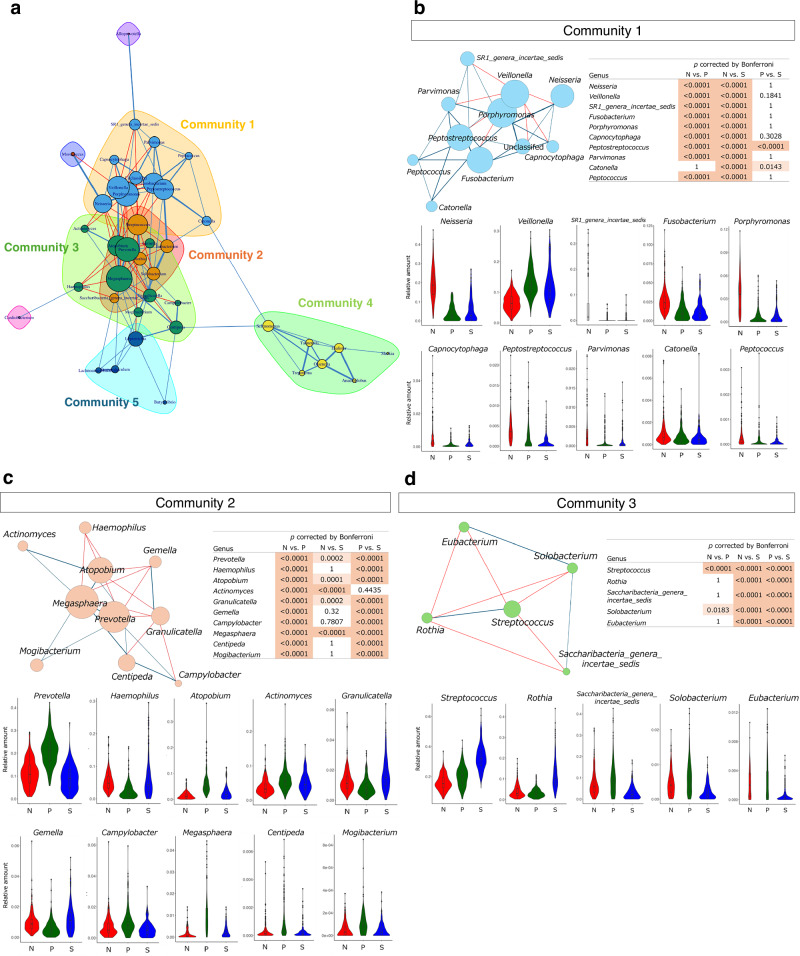
Co-occurrence network on the tongue microbiota. **a** Results of co-occurrence network and community detection. The colors of the nodes (bacterial genera) correspond to the communities. The size of each node is proportional to the number of nodes connected to it. The edge colors were set to blue or red for positive or negative correlations, respectively, with edge thickness corresponding to the absolute value of the correlation coefficient. **b**–**d** Co-occurrence network in Communities 1–3 and violin plots of the relative abundance of each bacterial genus by orotype. *P*-values were calculated using the Wilcoxon rank sum test with Bonferroni correction.

We hypothesized that each orotype would be shaped and characterized by co-occurring bacterial communities. To test this, we performed a co-occurrence network analysis, a method for visualizing pairs of bacterial genera with strong occurrence correlations^20^. The criteria were an absolute correlation coefficient >0.3 and a *q* < 0.05. The tongue microbiota formed a highly complex co-occurrence network (Fig. 3a).

Subsequently, community detection was conducted to identify the groups of bacterial genera that were more densely connected to each other than to the rest of the network. Eight communities were detected, five of which constituted more than two bacterial genera (Fig. 3a). *Neisseria*, *Prevotella*, and *Streptococcus* were assigned to Communities 1–3, respectively (Fig. 3b–d), suggesting that the bacterial genera in each community play a key role in defining the corresponding orotype. Notably, many bacterial genera in Community 1 were characteristically abundant or depleted in the N type. Similarly, Communities 2 and 3 were dominated by genera characteristic of the P and S types, respectively. The bacterial genera in Community 4 showed significant associations across the orotypes; however, these associations were not specific to a given orotype (Supplementary Fig. 3a). A similar pattern was observed in Community 5 (Supplementary Fig. 3b).

In Community 1, the largest number of edges was observed in *Porphyromonas*, *Fusobacterium*, and *Peptostreptococcus*, but not in *Neisseria*. *Atopobium* and *Megasphaera*, along with *Prevotella*, had a high number of edges in Community 2. In Community 3, the number of edges was relatively uniform across all genera.

### Association between orotypes and lifestyle factors, health status, and gut microbiota

To investigate the association between orotypes and lifestyle factors, data from 644 participants with complete information for relevant variables were analyzed (Fig. 1). Lifestyle characteristics grouped by orotype are shown in Supplementary Table 2. Age and medical history were not significantly associated with orotype, whereas sex, educational background, household composition, dietary habits, and oral care habits were. However, these associations were not adjusted for mutual confounding variables. To identify the lifestyle characteristics associated with orotypes after adjustment, variable selection using the filter method was performed, followed by adjusted logistic regression analysis.

**Fig. 4:**
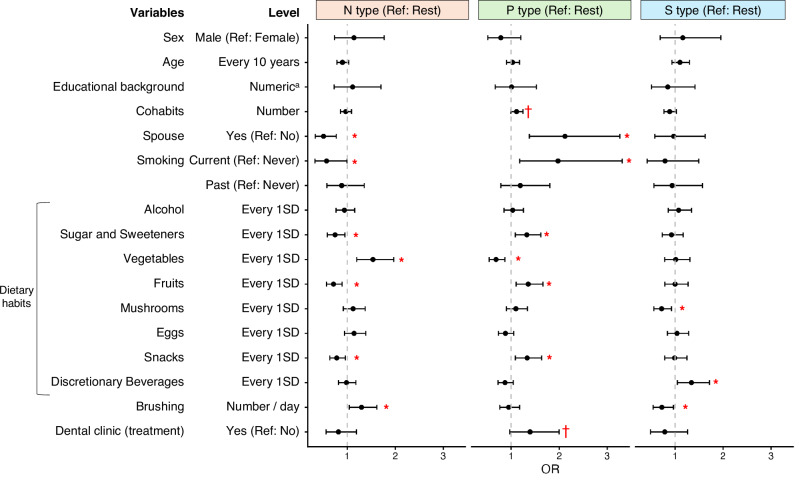
Associations between orotypes and lifestyle factors. Adjusted logistic regression analysis results (One-vs.-Rest). Sixteen variables were selected by variable selection and used as exposures. Dots and whiskers represent the odds ratios and 95% confidence intervals, respectively. *p < 0.05, †*p* < 0.1, SD standard deviation. ^a^Converted into the following numerical categories for analysis: 1, primary school or junior high school; 2, high school; 3, technical school; 4, college.

To avoid excessive variable reduction, a liberal threshold with *p* < 0.1 was applied to the results from orotype characterization (Supplementary Table 2), resulting in the selection of 16 out of 32 variables (Supplementary Table 3). Subsequently, logistic regression analysis was conducted with mutual adjustment for the 16 selected variables. The N type was characterized by being unmarried; non-smoking; a higher intake of vegetables; lower intake of sugar and sweeteners, fruits, and snacks; and more frequent tooth brushing (Fig. 4, Supplementary Table 4). The P type was characterized by being married; smoking; a higher intake of sugar and sweeteners, fruits, and snacks; and a lower intake of vegetables. The S type was characterized by a higher intake of discretionary beverages, a lower intake of mushrooms, and less frequent tooth brushing. These results highlight that not only oral care habits, but also other lifestyle factors are associated with orotypes.

**Fig. 5:**
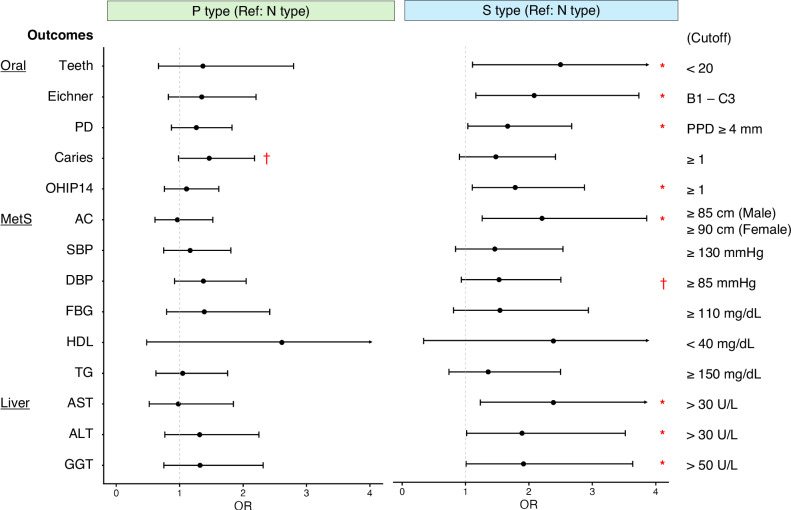
Associations between orotypes and health status. Odds ratios (ORs) for indicators outside the reference range were determined using adjusted logistic regression analysis. The N type was used as a reference. Age, sex, and nine variables associated with the orotypes (spouse, smoking, intakes of sugar and sweeteners, vegetables, fruits, mushrooms, snacks and discretionary beverages, and number of brushing) were used as confounders. Dots and whiskers represent the odds ratios and 95% confidence intervals, respectively. Teeth number of teeth, PD periodontal disease, OHIP14 Oral Health Impact Profile-14, AC abdominal circumference, SBP systolic blood pressure, DBP diastolic blood pressure, FBG fasting blood glucose, HDL high density lipoprotein (cholesterol), AST aspartate aminotransferase, ALT alanine aminotransferase, GGT gamma-glutamyl transferase, PPD probing pocket depth, MetS metabolic syndrome. *p < 0.05, †*p* < 0.1.

Subsequently, the relationships between health status and orotypes were analyzed. The population with the N type exhibited healthier oral conditions, including a greater number of teeth, fewer dental caries, and a lower prevalence of periodontal disease (Supplementary Table 5). Accordingly, the N type was designated as the reference orotype in the adjusted logistic regression analysis. As a result, not only oral conditions but also several biomarkers were associated with orotypes, even after adjusting for confounders such as age, sex, and nine variables associated with the orotypes, as shown in Fig. 4 (Fig. 5, Supplementary Table 6). Specifically, compared to the N type, the S type had higher odds ratios for being outside the reference range for eight indicators: number of teeth, Eichner classification, periodontal disease, Oral Health Impact Profile-14 (OHIP14)^34^, abdominal circumference, blood aspartate aminotransferase (AST), alanine aminotransferase (ALT), and gamma-glutamyl transferase (GGT). Similar results were obtained from the multiple regression analyses (Supplementary Table 6). Specifically, in addition to associations found with logistic regression analysis, multiple regression identified associations between the S type and a greater number of dental caries, reduced salivary flow, and elevated glycated hemoglobin (HbA1c) levels. The P type was associated with fewer teeth and reduced high-density lipoprotein (HDL) cholesterol levels. The S type showed significantly higher OHIP14 scores, particularly in subdomains 3 and 5–7, which reflect the psychological aspects of oral health. Furthermore, since it has been reported that oral health deterioration induces systemic inflammation, we analyzed the associations between orotypes and circulating inflammatory markers: high-sensitivity C-reactive protein (high-sensitivity CRP), interleukin-6 (IL-6), and tumor necrosis factor-alpha (TNF-α). However, the levels of these inflammatory markers were not elevated specifically in the S type (Supplementary Table 6).

Reportedly, oral and gut microbiomes can affect each other^35–38^. However, the orotypes showed no correlation with gut microbiome α-diversity (Supplementary Table 7). Furthermore, analysis of the relationship between orotypes and gut bacterial genera showed that only two gut bacterial genera, *Saccharibacteria* (genera Incertae Sedis) and *Actinomyces*, were significantly associated with the orotypes (Supplementary Table 7). Subsequently, based on previous studies^17,18^, we classified the gut microbiome types (enterotypes) and analyzed their associations with orotypes. While previous studies suggested three to five enterotypes, clustering into two groups provided the most distinct population separation in this study (Supplementary Fig. 4a, b). In the two-cluster model, *Bacteroides* and *Prevotella* were abundant in clusters 1 and 2, respectively. In the three-cluster model, *Ruminococcus, Prevotella*, and *Bacteroides* were abundant in clusters 1–3, respectively, similar to previous study findings^17^. Although no significant correlation was observed between the orotypes and enterotypes in the two-cluster model, a weak association was observed in the three-cluster model (*p* = 0.052, Supplementary Table 7). The P type, characterized by *Prevotella* abundance, exhibited a lower proportion of enterotypes with abundant *Prevotella* in the gut microbiome, suggesting that the tongue and gut microbiota do not necessarily share similar compositions.

### Construction of the orotype classification model

**Fig. 6:**
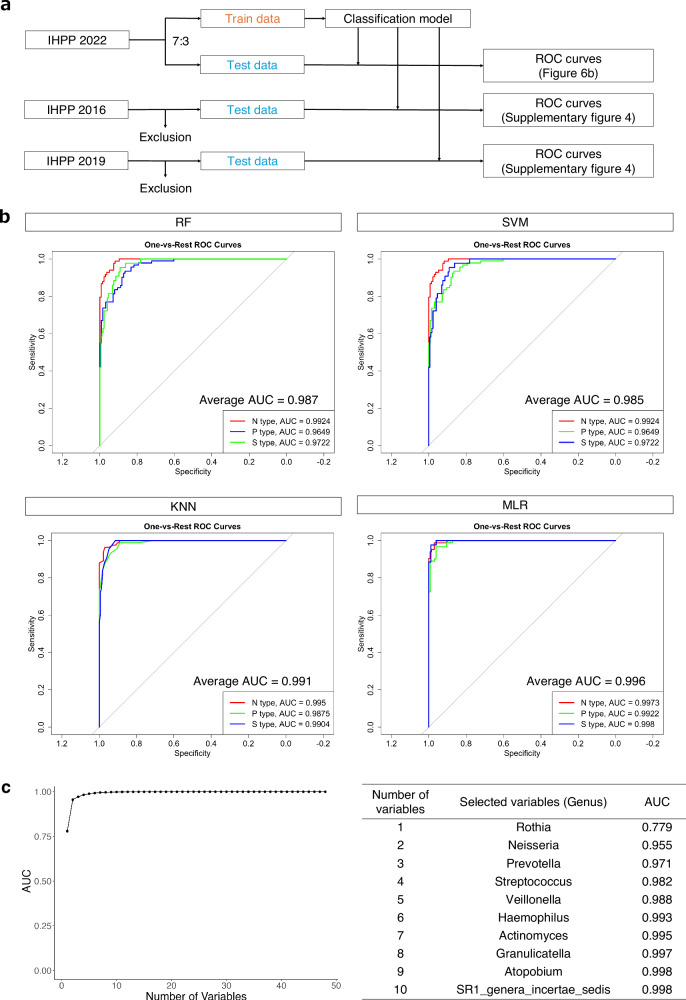
Orotype classification model construction. **a** Flowchart of classification model construction. Exclusion: Individuals who participated in 2022 IHPP. **b** Receiver operating characteristic (ROC) curve of the orotype classification model by the algorithm, RF Random Forest, SVM Support Vector Machine, KNN K Nearest Neighbors, MLR Multinomial Logistic Regression. The AUC for each orotype was calculated using the One-vs.-Rest method. **c** Change in AUC with variable reduction using stepwise backward selection. The selected variables are shown in the table on the right.

Before investigating the trajectories of these orotypes, a classification model was constructed using the 2022 tongue microbiota data, which were pre-split into training and test datasets, to establish a unified orotype classification (Fig. 6a). The optimal model was selected from four algorithms based on the area under the receiver operating characteristic curve (ROC–AUC) evaluated on the test dataset: random forest (RF), support vector machine (SVM), K-nearest neighbors (KNN), and multinomial logistic regression (MLR). As a result, the ROC–AUC was greater than 0.98 in all four algorithms (Fig. 6b). In particular, ROC–AUC was >0.99 in the MLR algorithm; therefore, this algorithm was adapted to the orotype classification model. The coefficients corresponding to each bacterial genus in the model are presented in Supplementary Table 8. The MLR-based classification model also showed a high ROC–AUC (>0.95) when applied to the IHPP datasets from different survey years (2016 and 2019), after excluding individuals who participated in the 2022 IHPP (Supplementary Fig. 5). Subsequently, stepwise backward selection was applied to evaluate the change in AUC with variable reduction. The AUC with only one variable (*Rothia*) was 0.779, while the AUC with two variables (*Rothia* and *Neisseria*) reached 0.955 (Fig. 6c).

### Orotype trajectory

**Fig. 7:**
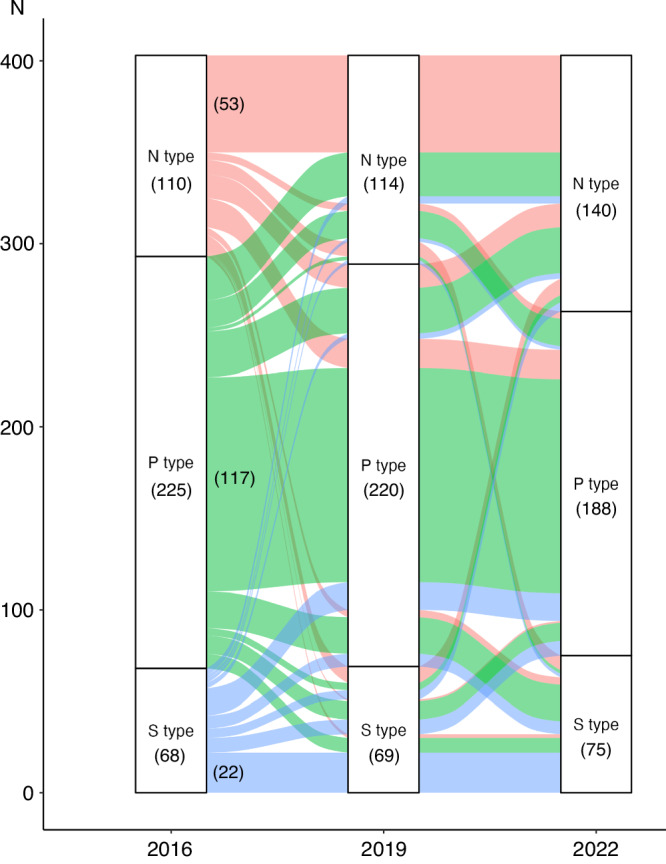
Orotype trajectory. Alluvial diagram illustrating the trajectories of individuals’ orotypes in 2016, 2019, and 2022.

To confirm orotype trajectory, data from 403 individuals participating in all three IHPP (2016, 2019, and 2022) were analyzed (Fig. 1). The orotype classification model was applied to tongue microbiota data from 2016, 2019, and 2022. The alluvial diagram in Fig. 7 illustrates the trajectory of an individual’s orotype. In 2016, 110 out of 403 participants were classified as N type, 53 of whom (48%) maintained the same orotype over a 6-year period. Likewise, 117 out of 225 participants (52%) maintained the P type and 22 out of 68 (32%) participants maintained the S type over the 6-year period. This indicates that the orotypes, especially the N and P types, were relatively stable. Interestingly, the distribution of orotypes remained stable in 2016 and 2019 but changed significantly by 2022.

### Associations between orotype transition and changes in lifestyle factors and health status

Using data from 2016 and 2019, in which the orotype proportions did not differ significantly, the relationship between the orotype transitions and changes in lifestyle factors or health status was investigated. First, to examine whether lifestyle changes influenced orotypes, we assessed the transition of orotypes across three groups categorized by changes in lifestyle indicators: decreased, stable, and increased. Consistent with the results of the cross-sectional analyses, the proportion of the N type increased in the group with increased vegetable intake; the proportion of the P type increased in the group with increased intake of sugar and sweeteners or snacks; and the proportion of the S type increased in the group with increased intake of discretionary beverages and decreased intake of mushrooms (Supplementary Fig. 6). We also examined changes in lifestyle factors and health status in the orotype transition groups (Supplementary Table 9). While a few variables, such as the intake of vegetables, sugar and sweeteners, and discretionary beverages, showed changes consistent with the results of the cross-sectional analyses, most variables did not differ significantly across the orotype transition groups. However, these results should be interpreted with caution, given the small sample size within each group and the presence of baseline differences among the groups.

## Discussion

In this study, tongue microbiota was classified into three distinct orotypes using data from IHPP participants living in northern Japan. These orotypes were designated as N, P, and S types, based on the characteristically abundant bacterial genera *Neisseria*, *Prevotella*, and *Streptococcus*, respectively (Fig. 2a). Each orotype formed a co-occurring community composed of five to ten bacterial genera (Fig. 3b–d). The orotypes were associated with several lifestyle factors and health status (Figs. 4 and 5). These findings emphasize the need for comprehensive evaluation of the tongue microbiota. In addition, as a high-performance classification model for orotypes was constructed, further studies are warranted to validate the generalizability of this classification and explore its associations with health conditions other than the ones already identified in this study. This approach is also expected to contribute to improvements in health monitoring and disease prediction models.

The number of orotypes to achieve the most distinct population separation was determined to be three, based on the CH Index (Supplementary Fig. 1a). In two previous studies involving Korean and Mexican individuals, the oral microbiome was divided into three clusters and the representative bacterial genera of each cluster were *Neisseria* (or *Fusobacterium*), *Prevotella*, and *Streptococcus*^15,16^. Although this study targeted a specific population from a particular region with similar genetic backgrounds and lifestyles, the robustness of the results is supported by these previous reports. In this study, the oral microbiota was quantified using tongue-coating samples, whereas previous studies involving Korean and Mexican populations used saliva samples and mouthwash samples, respectively. Despite these differences in sampling methods, the orotype classification was largely consistent. This may be because the microbiota of tongue coating and saliva are more similar compared with that of gingival plaque^39^.

Co-occurrence network analyses identified distinct bacterial communities characteristic of each orotype (Fig. 3). In Community 1, which was dominated by bacterial genera characteristic of the N type, *Porphyromonas* and *Peptostreptococcus* were central to the community structure despite their low relative abundance. This suggests that they may play a key role in the N type-specific bacterial community formation. Similarly, in Community 2, despite a relative abundance <1%, *Megasphaera* was positioned at the center of the community, along with *Prevotella*. An association between *Megasphaera* and dyslipidemia has been reported in previous studies^40^, supporting our observation of lower HDL cholesterol levels in the P type (Supplementary Table 6). In Community 3, all bacterial genera were equally connected to other bacterial genera. Taken together, the co-occurrence network analysis suggested core bacterial genera that may play a key role in shaping bacterial community. In this study, two communities other than the three mentioned above were detected (Supplementary Fig. 3). Both communities consisted of bacterial genera with a relative abundance <1%, suggesting that minor bacterial genera interact with each other.

We constructed a high-performance classification model with an AUC > 0.95 (Fig. 6b). As expected from the results of the co-occurrence network, this was attributed to the formation of bacterial communities, which reduced the noise among variables and enhanced the robustness of the classification model. Interestingly, a highly accurate classification model was constructed using only two bacterial genera (*Rothia* and *Neisseria*) (Fig. 6b). This suggests the potential for inexpensive and simple identification of an individual’s orotype, which may facilitate its application in healthcare. Furthermore, the coefficients in the classification model were the characteristics of each orotype (Supplementary Table 8). Specifically, the bacterial genera in Community 1 had large absolute coefficient values for both P and S types, indicating that they are important variables for distinguishing the N type. Similarly, those in Communities 2 and 3 had large absolute coefficient values for only the P and S types, respectively, indicating that they are important variables for distinguishing between the P and S types. In addition, the bacterial genera with large absolute coefficient values overlapped with those located at the center of the communities. Taken together, the results of the classification model were strongly associated with those of the co-occurrence network analysis, mutually reinforcing the validity of both approaches.

These orotypes were associated with several lifestyle factors (Fig. 4). Smoking has been reported to greatly influence the oral microbiome through several mechanisms, including salivary acidosis, reduced oxidative capacity, and modulation of host immunity^25,26^. Specifically, the relative abundance of *Prevotella* and *Veillonella* increased, whereas that of *Neisseria*, *Granulicatella, Peptostreptococcus*, and *Gemella* decreased, consistent with the characteristics of the N type (less smoking) and P type (more smoking). Regarding household composition, individuals in the P type had spouses and lived with more cohabitants. It has been reported that the oral microbiome is shared among household members^41,42^. The P type bacterial community may have a stronger dominance than that of the other orotypes. While familial relationships among individuals were not identified in this study, it is possible that individuals living in the same household share the same orotype. Regarding oral care, infrequent brushing was associated with the S type (Fig. 4), which is plausible given that *Streptococcus* is a representative biofilm-forming bacterial genus.

Regarding dietary habits, different food intake levels for items such as sugar and sweeteners, vegetables, fruits, mushrooms, snacks, and discretionary beverages, were associated with different orotypes, suggesting that the carbohydrates quality may influence the oral microbiome. At the nutrient level, the N type was characterized by low monosaccharide intake and high dietary fiber intake, the S type showed high monosaccharide intake but low dietary fiber intake, and the P type exhibited high intakes of both (Supplementary Table 10). These findings are consistent with the known metabolic capacities of *Prevotella* for dietary fiber and *Streptococcus* for monosaccharides^16,43,44^. In contrast, *Neisseria* exhibits a low ability to metabolize dietary fiber, which is inconsistent with the current findings. This discrepancy may be explained by *Neisseria*’s symbiotic interactions with other bacterial genera. Although alcohol consumption has been reported to influence the oral microbiome^29,32^, these relationships were not observed in our study. The impact of alcohol consumption may be localized and insufficient to cause an orotype shift.

The orotypes in our study were associated not only with oral health but also with systemic biomarkers. Since this study used tongue-coating samples, not plaque samples, it is difficult to mention the causal relationship and mechanism between tongue microbiota and oral disorder onset. Regarding systemic biomarkers, these relationships involve systemic inflammation exacerbated by local inflammation due to periodontitis, as well as bacterial invasion into the oral blood vessels and digestive tract^2,3,15,45–48^. However, the orotypes in this study were not associated with inflammation-related biomarkers such as high-sensitive CRP, IL-6, and TNF-α. Taken together, although the mechanisms by which orotypes are associated with oral health and systemic biomarkers remain unclear, our findings suggest that orotypes may serve as markers of systemic health status. The use of simpler tongue-coating sampling may enable health status monitoring and thereby lower the barriers to practical and societal implementation.

The oral and gut microbiomes are reportedly associated^35–38^. However, we found only two gut bacterial genera associated with orotypes, and no association of orotypes with enterotypes or α-diversity of the gut microbiome. This may be attributed to the fact that the participants in this study were relatively healthy adults. Although oral bacteria can invade the digestive tract due to impaired barrier function in patients or infants, such cases are infrequently observed in healthy adults.

Approximately half of our study participants maintained the same orotype for over 6 years (Fig. 7). A previous study showed that intra-individual variation over time in the oral microbiota is smaller than inter-individual variations and that bacterial genera with high relative abundance exhibit particularly low variability^39^. Given that bacterial genera with high relative abundance significantly contributed to orotype classification in this study, the orotype stability in this study is plausible. In addition, the proportion of orotypes in 2022 was different from that in 2016 and 2019, suggesting that lifestyle changes due to the COVID-19 pandemic might have influenced the tongue microbiota. Specifically, the observed increase in the proportion of the N type may be attributable to improvements in lifestyle-related behaviors. Moreover, pandemic-related factors, including mask wearing and reduced interpersonal contact, may also have contributed to changes in oral microbiota composition. Indeed, previous studies have reported that individuals who share common spaces, such as senior citizens’ centers and universities, tend to exhibit more similar oral microbiota profiles^49,50^.

This study has some limitations. This study included a population from a specific region in Japan sharing similar genetic backgrounds and lifestyles. Further studies are warranted to validate the generalizability of the orotype classification provided herein. In addition, some health indicators were assessed only using crude measures (i.e. periodontal diseases: probing pocket depth ≥4 mm), which did not allow us to capture the degree of severity. Analyses using continuous variables are warranted.

In conclusion, We classified the tongue microbiota into three types (orotypes), each exhibiting a distinct bacterial co-occurrence network structure and specific associations with lifestyle factors, including smoking, and dietary and oral care habits. The S type was associated with higher odds of abnormal oral health and metabolic syndrome-related outcomes compared to the N type. Furthermore, we developed a robust classification model to predict orotypes (ROC–AUC > 0.95). These findings highlight the value of orotype classification for monitoring tongue microbiota communities and suggest its potential in health risk assessment.

## Methods

### Study design

This study included participants from the IHPP^51^. This is a health checkup program targeting men and women aged ≥20 years living in northern Japan. Data from 2022 were used for cross-sectional analyses, and data from 2016, 2019, and 2022 were used for longitudinal analyses (Fig. 1). Oral microbiota data were obtained from tongue-coating samples. Information on demographic data and lifestyle was collected using questionnaires. Health status indicators were measured from blood samples or medical checkups. Gut microbiome data were obtained from fecal samples.

Data collection for this study was approved by the Ethics Committee of Hirosaki University School of Medicine (approval numbers: 2016-028, 2020-046, 2021-166) and was conducted in accordance with the principles of the Declaration of Helsinki. Written informed consent was obtained from all participants before their inclusion in this study. This study’s data analysis was approved by the Ethics Committee of Hirosaki COI-NEXT (approval number: HCN2024-20-0926). An opt-out procedure for data analysis was preliminarily conducted, and participants were allowed to opt out at any time, even after the analysis began.

### Microbiota analysis and data processing

Tongue microbiota data were obtained following previously reported methods^27^. Briefly, tongue coating samples were collected, before breakfast and tooth brushing, at the health check-up. The collected samples were stored at 4 °C in tubes containing a mixed solution (4 M guanidium thiocyanate, 100 mM Tris-HCl [pH 8.0], 40 mM EDTA, and 0.001% bromothymol blue) until analysis. DNA was extracted from the bead-processed samples (2016) using an automated nucleic acid extraction system (Precision System Science, Chiba, Japan) with a MagDEA DNA 200 (GC) reagent kit (Precision System Science). DNA was extracted from the bead-processed samples (2019, 2022) using an automated nucleic acid extraction system GENE PREP STAR PI-480 (Kurabo Industries, Osaka, Japan) with a reagent kit NR-201 (Kurabo Industries). The V3–V4 region of the 16S rRNA gene was amplified using a universal primer set, according to a previous report^52^. Sequencing was performed using the Illumina MiSeq system, and quality control, trimming, merging, and chimera detection were performed using DADA2^53^. Bacterial taxonomy was assigned using Expanded Human Oral Microbiome Database^54^.

To obtain gut microbiome data, we followed previous studies^55^. Briefly, fecal samples collected from 3 days before up to the day of the health check-up were used. The samples were stored at ≤4 °C in a commercially available fecal collection kit Metabolokeeper® (TechnoSuruga Laboratory Co., Ltd., Shizuoka, Japan) until analysis. DNA extraction and subsequent procedures performed on these samples were the same as those used for the tongue microbiota samples, except that bacterial taxonomy was assigned using Ribosomal Database Project version 16^56^.

Data preprocessing involved the combination of bacterial genera with a detection rate of ≤50% into a single category labeled “Other genera.” The detection rate was defined as the proportion of participants with at least one read of the bacterial genus among all analyzed subjects. As a result, a total of 48 variables were used in the analyses of the tongue microbiota, including 46 bacterial genera with a detection rate above 50%, “Other genera”, and “Unclassified”. The Jensen–Shannon distance was used as a distance metric. The R package “philentropy” was used for this calculation.

### Microbiome typing and co-occurrence analysis

For orotype and enterotype classification, we followed previously reported methods^17,18^. Briefly, the optimal number of clusters was determined based on the Calinski–Harabasz (CH) index^33^, and clustering was performed using Partitioning Around Medoids (PAM). The R packages “cluster” and “fpc” were used for these analyses.

Co-occurrence network analysis was performed following previously described procedures^57,58^. Briefly, we first calculated the correlation coefficients of relative abundances between bacterial genera using Compositionality Corrected by REnormalization and PErmutation (CCREPE), which is based on Spearman’s correlation coefficient, although it calculates *p*-values under the constraint that the sum of the relative abundances of the bacterial genera equals 1^57^. This method is effective for computing correlations between compositional data, such as microbiome data. Subsequently, a network diagram was visualized using edges that met the criteria of |ρ| > 0.3 and q < 0.05. Nodes were positioned based on a force-directed layout algorithm. The size of each node was proportional to the number of its connections with other nodes. The edge colors were set to blue or red for positive or negative correlations, respectively, with edge thickness corresponding to the absolute value of the correlation coefficient. Community detection was performed using the edge betweenness method. The R packages “CCREPE” and “igraph” were used for these analyses.

α-diversity was calculated by applying the Shannon index and Simpson index. The R package “vegan” was used for this analysis.

### Orotype classification model construction

First, the data were split into training and test sets in a 7:3 ratio. Using the training set, candidate models were constructed using four algorithms: Random Forest (RF), Support Vector Machine (SVM), K-Nearest Neighbor (KNN), and multinomial logistic regression (MLR). Subsequently, the performance of each model was validated based on the area under the receiver operating characteristic curve (ROC–AUC). Hyperparameters were set as follows: for the SVM, a linear kernel was chosen based on the ROC–AUC among linear, polynomial, radial basis function, and sigmoid kernels; for the RF, the optimal number of variables randomly selected at each split was determined to be 12 based on the results of hyperparameter tuning; and for the KNN, the optimal number of neighbors (k) was determined to be 21 based on the results of hyperparameter tuning.

The R packages “randomForest,” “e1071,” “caret,” “nnet,” and “pROC” were used for these analyses.

### Questionnaires

The participants were asked questions on the following aspects: demographic information, lifestyle factors (smoking, drinking, sleeping, exercise, and oral care), dietary habits, and medical history. The details are presented in Supplementary Table 11.

Dietary habits were assessed using the 172-item long-food frequency questionnaire (long-FFQ) developed for the Japan Public Health Center-based prospective Study for the Next Generation (JPHC-NEXT)^59^. Food and nutrient intake were calculated using FFQ NEXT (Kenpakusha, Tokyo, Japan) based on the Standard Tables of Food Composition in Japan 2020 (eighth revised edition). In 2016 and 2019, the Brief-type Self-administered Diet History Questionnaire (BDHQ) was used^60,61^.

### Measurements of health states

The following biomarkers and variables were measured: number of teeth, Eichner classification, periodontal disease, number of dental caries, Oral Health Impact Profile (OHIP14), calculus, salivary IgA, salivary secretion, metabolic syndrome-related biomarkers, liver function-related biomarkers, and inflammation-related biomarkers. Detailed related information is reported in Supplementary Table 11.

### Statistical analysis

To analyze the associations between orotypes and the relative abundance of bacterial genera or α-diversity, the Wilcoxon rank sum test with Bonferroni correction was performed. To analyze the associations between orotypes and lifestyle factors, multinomial logistic regression (One-vs.-Rest) was performed, treating lifestyle factors as exposures and orotype as the outcome. To analyze the associations between orotypes and health status, multinomial logistic regression and multiple regression (reference: N type) were performed, treating orotype as the exposure and health status as the outcomes. Statistical significance was set at *p* < 0.05. All analyses were performed using R software (version 4.4.2).

## Supplementary information

Supplementary information

Supplementary data

Supplementary tables

## Data Availability

All data in this study are available upon request from the Hirosaki University COI Program Institutional Data Access/Ethics Committee (contact via e-mail: coi@hirosaki-u.ac.jp) for researchers eligible for data access. The data cannot be shared publicly because of ethical concerns.
