# Supplementary material for: Classification of the tongue microbiota and its associations with lifestyle factors and health status
Source: NPJ Biofilms Microbiomes. 2026 Feb 26;12:75. doi: 10.1038/s41522-026-00936-6 (PMC13061929; doi:10.1038/s41522-026-00936-6)
Supplement: Supplementary file 1 — Supplementary information [file 41522_2026_936_MOESM1_ESM.pdf]

I. Supplementary Figures

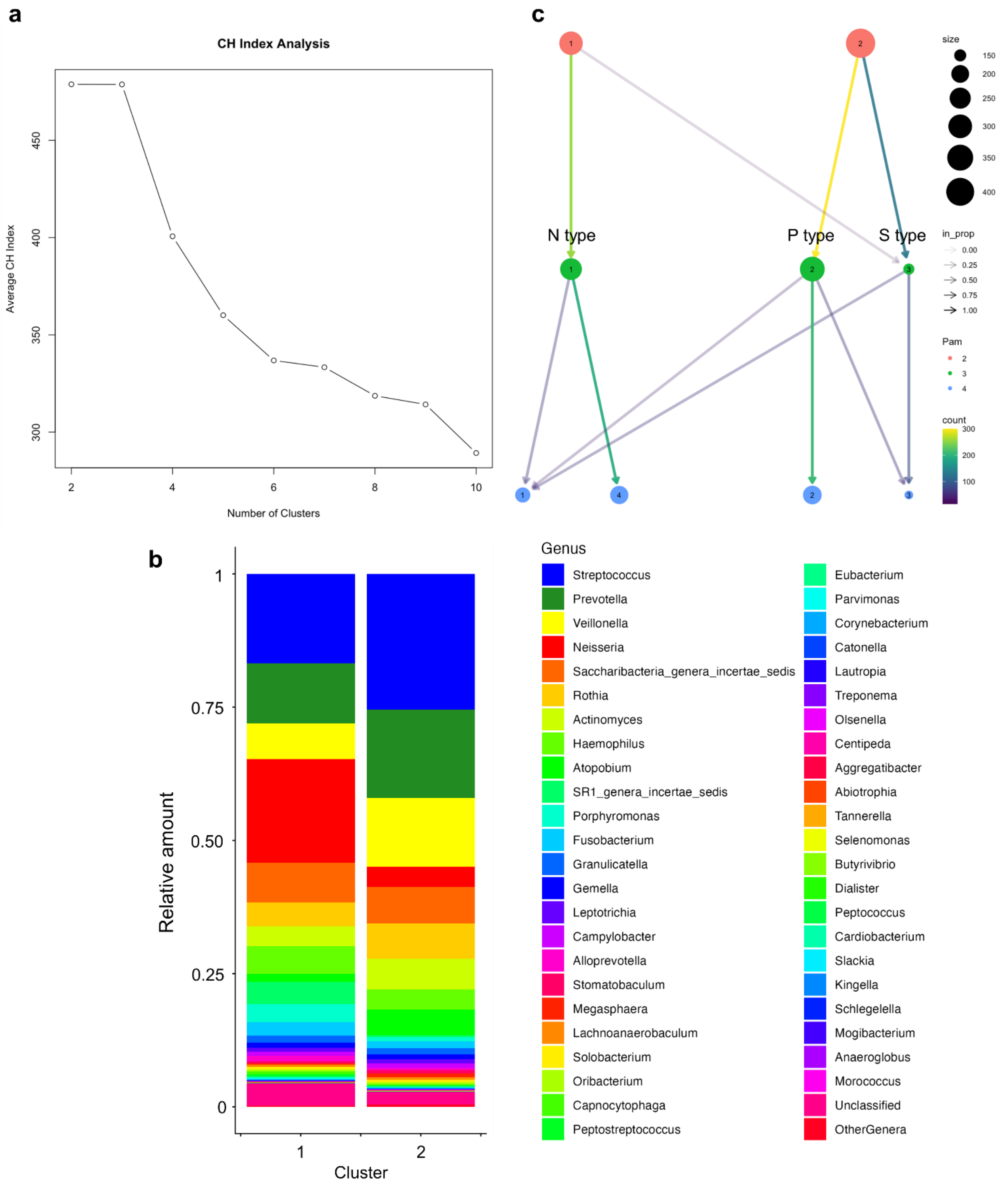

Supplementary Fig. 1: Examination of the number of orotypes

a. CH Index results for tongue microbiota clustering

b. Stacked bar plots of the relative abundance of tongue bacterial genera in a two-clustering model

c. Transition diagram of the individual’s orotype across two- to four-clustering models

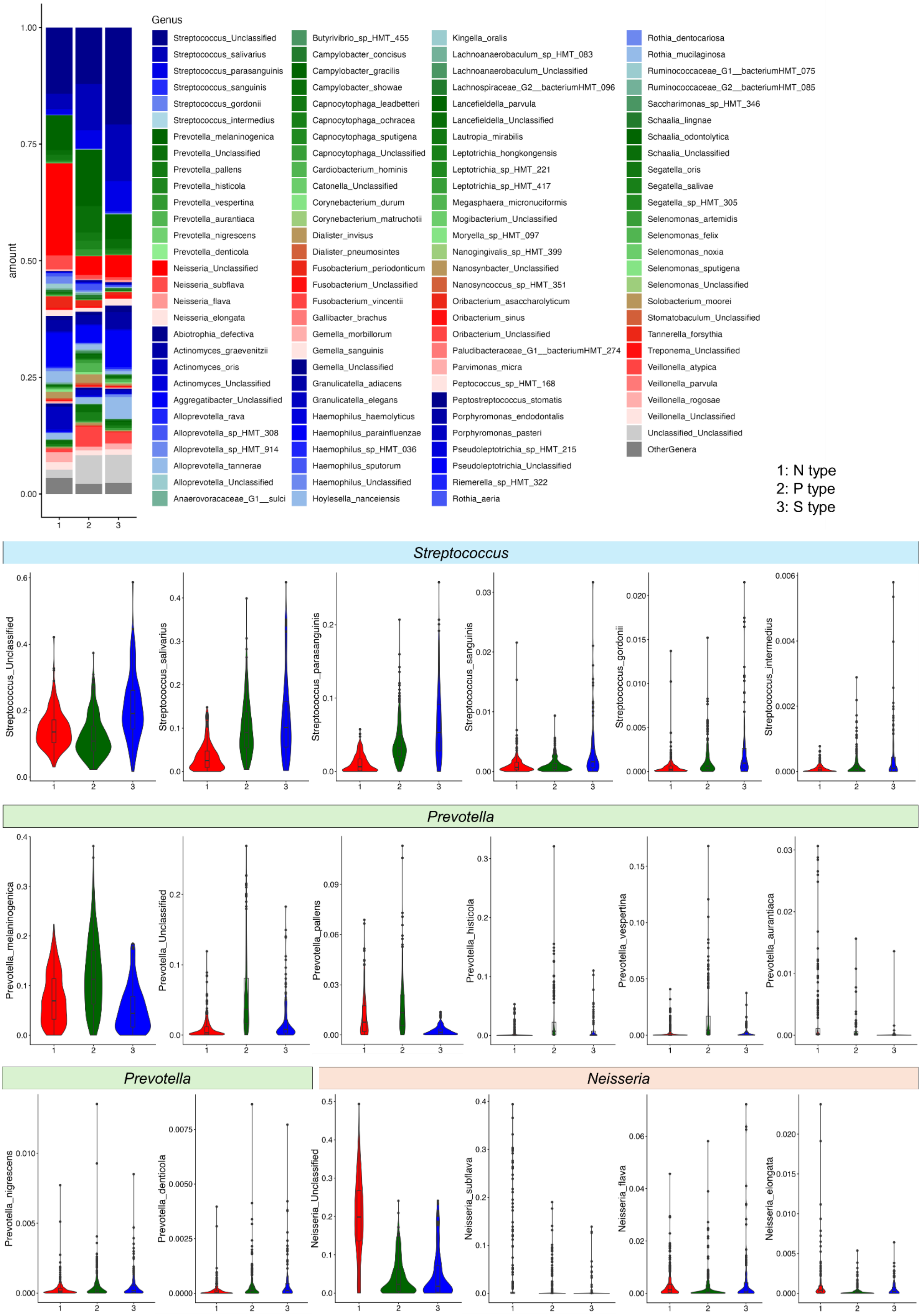

Supplementary Fig. 2: Relative abundance of tongue bacterial species by orotypes

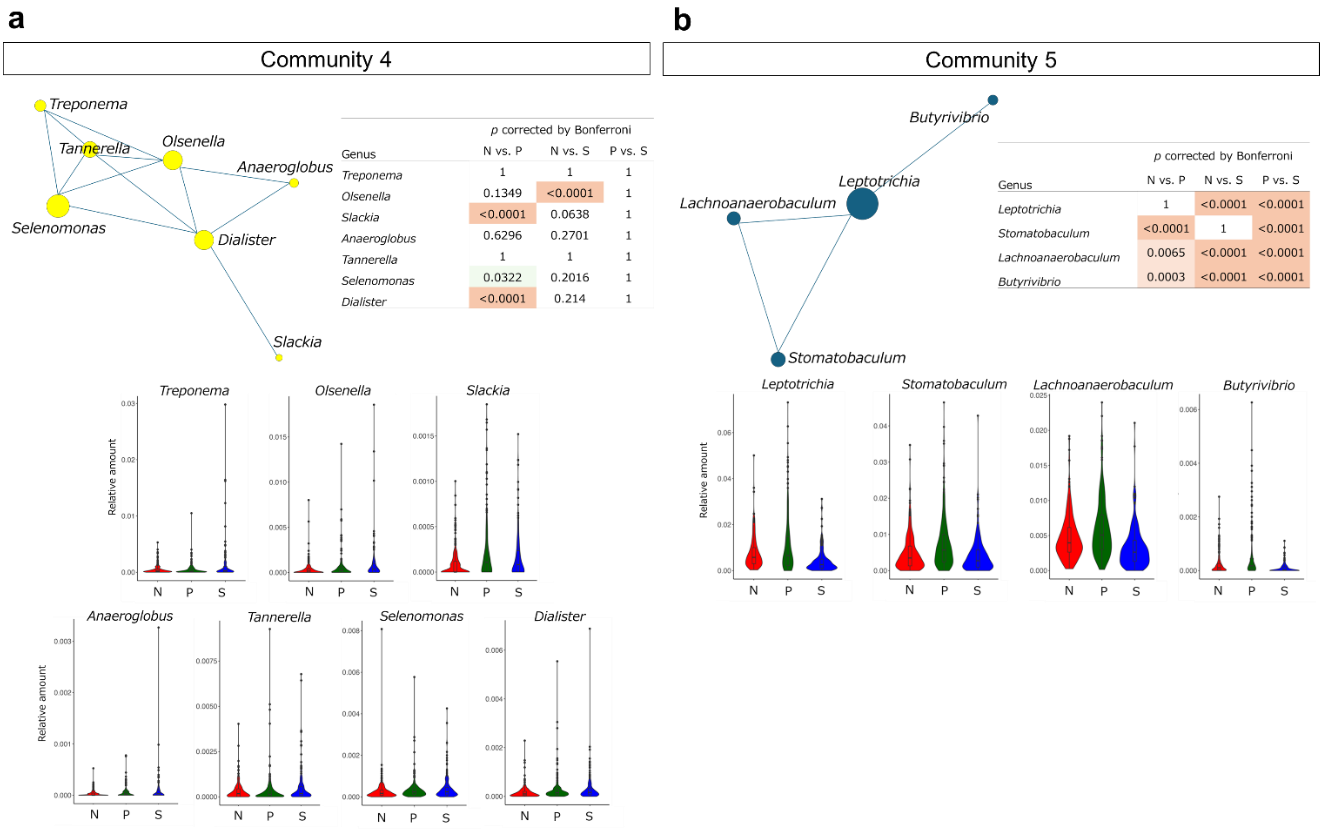

Genus	*p* corrected by Bonferroni N vs. P	N vs. S	P vs. S
Treponema	1	1	1
Olsenella	0.1349	<0.0001	1
Slackia	<0.0001	0.0638	1
Anaeroglobus	0.6296	0.2701	1
Tannerella	1	1	1
Selenomonas	0.0322	0.2016	1
Dialister	<0.0001	0.214	1

Genus	*p* corrected by Bonferroni N vs. P	N vs. S	P vs. S
Leptotrichia	1	<0.0001	<0.0001
Stomatobaculum	<0.0001	1	<0.0001
Lachnoaerobaculum	0.0065	<0.0001	<0.0001
Butyrivibrio	0.0003	<0.0001	<0.0001

Supplementary Fig. 3: Co-occurrence network in tongue bacterial Communities 4 and 5

a, b. Co-occurrence network in Communities 4 and 5, and violin plots of the relative abundance of each bacterial genus by orotype. P-values were calculated using the Wilcoxon rank-sum test with Bonferroni correction.

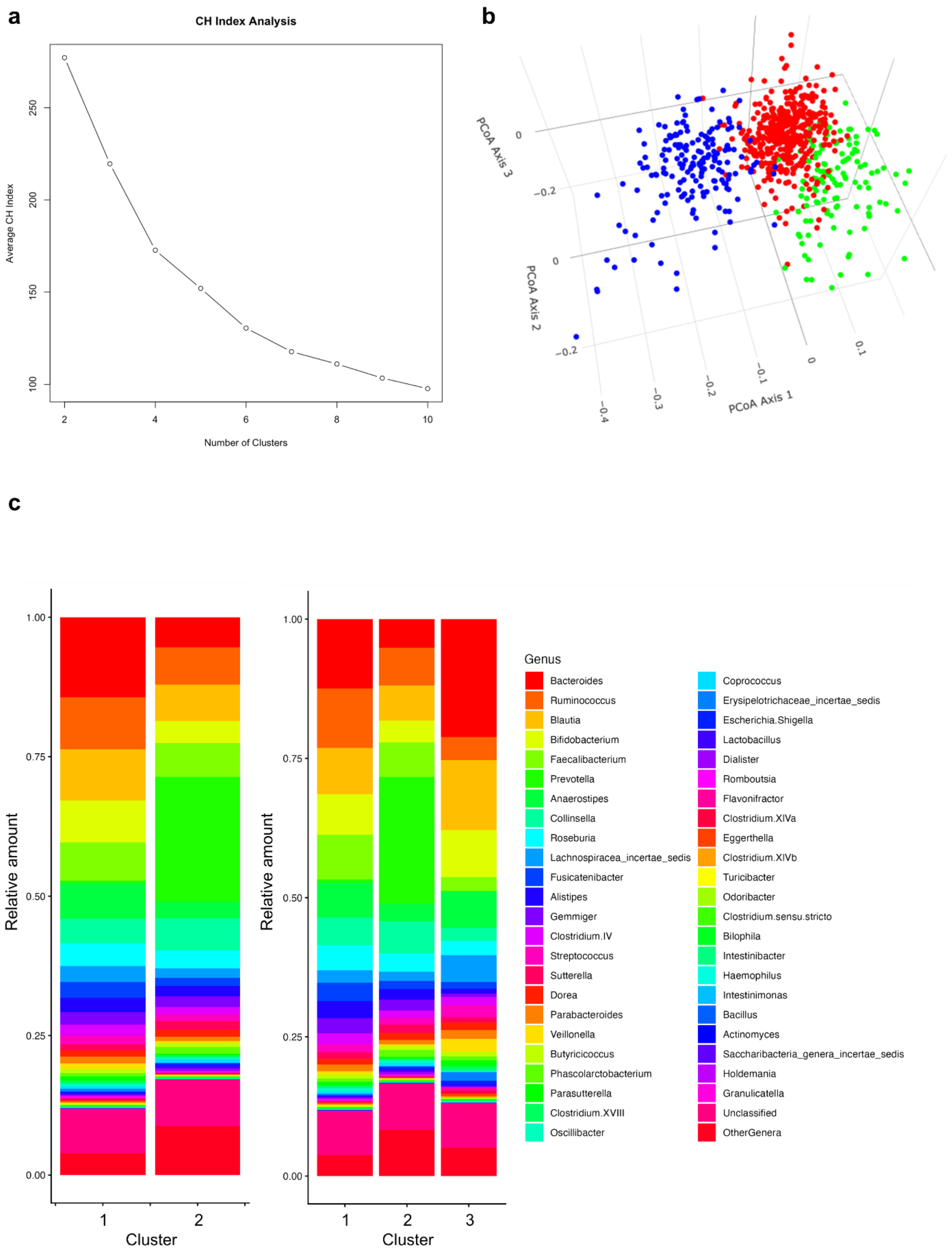

Supplementary Fig. 4: Classification of the gut microbiome (enterotype)

a. CH Index result for gut microbiome clustering

b. Distribution of the gut microbiome using Principal Coordinates Analysis (PCoA). Plot colors represent specific enterotypes.

c. Stacked bar plots of the relative abundance of gut bacterial genera in two- and three-clustering models

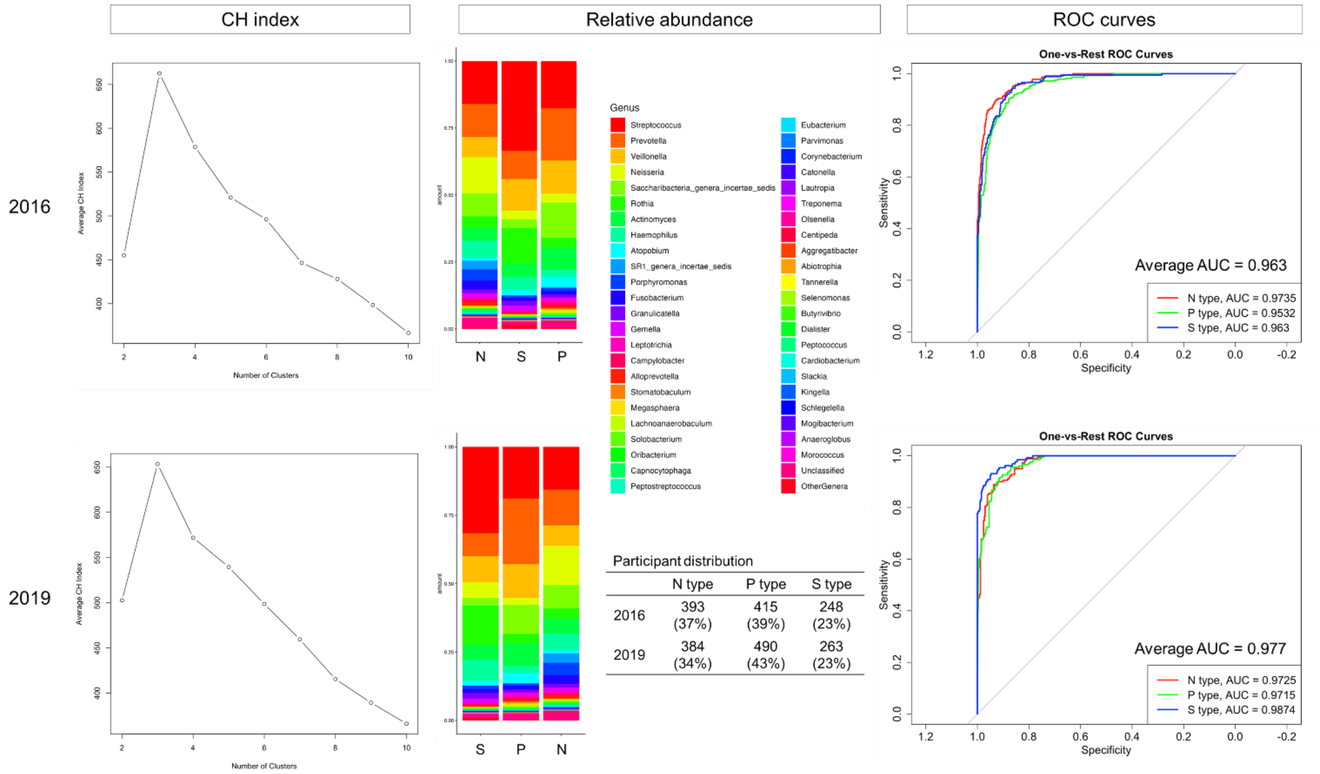

Supplementary Fig. 5: Validation of classification model using 2016 and 2019 IHPP data

The data from each year were classified into three clusters based on the results of the CH index. As with the 2022 data, all clusters were categorized into N-, P-, and S-types, which were used as reference labels for model validation. The classification performance of the MLR-based model was shown using ROC curves.

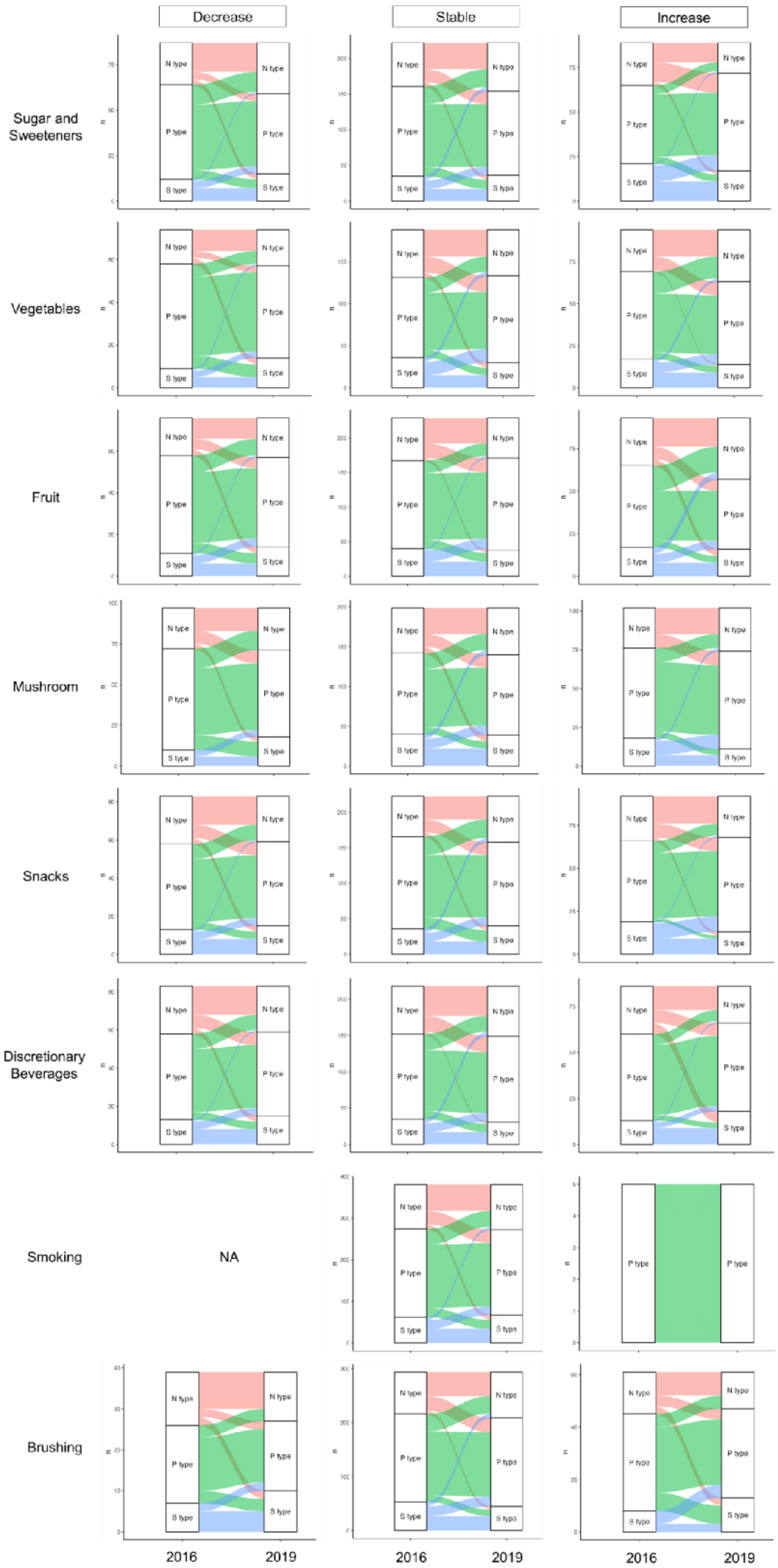

Supplementary Fig. 6: Transition of the orotype across three groups categorized by changes in lifestyle factors

Dietary intake levels were divided into annual tertiles, and participants were grouped based on changes between tertiles over time.

Smoking status was categorized as “Decrease” if the participant had quit smoking, “Increase” if they had started smoking, and “Stable” if there was no change.

The number of brushing sessions was categorized into groups based on an increase or decrease in the number of brushing sessions per day.

II. Supplementary Tables

Supplementary Data 1: α-diversity and relative abundances of genera and species in the tongue microbiome by orotype

Supplementary Data 2: Characteristics by orotype

Supplementary Data 3: Result of variable selection

Supplementary Data 4: Associations between orotypes and lifestyle factors by logistic regression analysis

Supplementary Data 5: Health status by orotype

Supplementary Data 6: Associations between orotypes and health status by logistic regression analysis and multiple regression analysis

Supplementary Data 7: α-diversity, type (enterotype) and genera in the gut microbiome by orotype

Supplementary Data 8: Coeffficients corresponding to each bacterial genus in the orotype classification model

Supplementary Data 9: Associations between the transition of orotypes and the change of lifestyle factors and health status

Supplementary Data 10: Nutrient intakes by orotype

Supplementary Data 11: List of measurement items and its methods
