# Supplementary material for: Classification of the tongue microbiota and its associations with lifestyle factors and health status
Source: NPJ Biofilms Microbiomes. 2026 Feb 26;12:75. doi: 10.1038/s41522-026-00936-6 (PMC13061929; doi:10.1038/s41522-026-00936-6)
Supplement: Supplementary file 3 — Supplementary tables [file 41522_2026_936_MOESM3_ESM.docx]

Supplementary Table 1: α-diversity and relative abundances of genera and species in the tongue microbiome by orotype

| Category | Variables | Unit | N type | P type | S type | P value | | |
| --- | --- | --- | --- | --- | --- | --- | --- | --- |
|  | n |  | 261 | 322 | 146 | N vs. P | N vs. S | P vs. S |
| α-diversity | Shannon |  | 2.48 ± 0.18 | 2.34 ± 0.21 | 2.13 ± 0.28 | <0.001 | <0.001 | <0.001 |
|  | Simpson |  | 0.87 ± 0.03 | 0.85 ± 0.03 | 0.81 ± 0.06 | <0.001 | <0.001 | <0.001 |
| Genus | *Streptococcus* | % | 16.22 ± 5.65 | 21.42 ± 7.27 | 33.38 ± 9.19 | <0.001 | <0.001 | <0.001 |
|  | *Prevotella* | % | 10.82 ± 5.46 | 20.26 ± 6.70 | 8.09 ± 4.88 | <0.001 | <0.001 | <0.001 |
|  | *Veillonella* | % | 6.55 ± 2.79 | 12.85 ± 4.35 | 11.76 ± 5.94 | <0.001 | <0.001 | 0.184 |
|  | *Neisseria* | % | 20.13 ± 8.19 | 4.33 ± 4.26 | 5.04 ± 5.86 | <0.001 | <0.001 | 1.000 |
|  | *Saccharibacteria_genera_incertae_sedis* | % | 7.39 ± 6.07 | 8.83 ± 6.97 | 2.68 ± 2.90 | 1.000 | <0.001 | <0.001 |
|  | *Rothia* | % | 4.02 ± 3.04 | 3.50 ± 2.40 | 13.96 ± 7.43 | 1.000 | <0.001 | <0.001 |
|  | *Actinomyces* | % | 3.56 ± 1.94 | 5.94 ± 3.10 | 5.13 ± 2.95 | <0.001 | <0.001 | 0.444 |
|  | *Haemophilus* | % | 5.43 ± 3.13 | 2.71 ± 2.41 | 5.94 ± 5.61 | <0.001 | 1.000 | <0.001 |
|  | *Atopobium* | % | 1.41 ± 1.30 | 5.58 ± 4.01 | 2.66 ± 2.69 | <0.001 | <0.001 | <0.001 |
|  | *SR1_genera_incertae_sedis* | % | 4.69 ± 6.94 | 0.46 ± 1.33 | 0.11 ± 0.43 | <0.001 | <0.001 | 1.000 |
|  | *Porphyromonas* | % | 3.59 ± 2.26 | 0.85 ± 1.27 | 0.76 ± 1.05 | <0.001 | <0.001 | 1.000 |
|  | *Fusobacterium* | % | 2.62 ± 1.64 | 1.37 ± 1.22 | 1.28 ± 1.27 | <0.001 | <0.001 | 1.000 |
|  | *Granulicatella* | % | 1.24 ± 0.71 | 0.96 ± 0.65 | 1.77 ± 1.12 | <0.001 | <0.001 | <0.001 |
|  | *Gemella* | % | 0.97 ± 0.64 | 0.68 ± 0.57 | 1.27 ± 0.95 | <0.001 | 0.320 | <0.001 |
|  | *Leptotrichia* | % | 0.75 ± 0.68 | 1.02 ± 1.13 | 0.42 ± 0.51 | 1.000 | <0.001 | <0.001 |
|  | *Campylobacter* | % | 0.69 ± 0.60 | 0.99 ± 0.78 | 0.53 ± 0.47 | <0.001 | 0.781 | <0.001 |
|  | *Alloprevotella* | % | 1.12 ± 2.03 | 0.47 ± 0.78 | 0.22 ± 1.06 | <0.001 | <0.001 | <0.001 |
|  | *Stomatobaculum* | % | 0.47 ± 0.51 | 0.72 ± 0.70 | 0.38 ± 0.42 | <0.001 | 1.000 | <0.001 |
|  | *Megasphaera* | % | 0.14 ± 0.19 | 0.87 ± 0.76 | 0.25 ± 0.28 | <0.001 | <0.001 | <0.001 |
|  | *Lachnoanaerobaculum* | % | 0.45 ± 0.31 | 0.59 ± 0.41 | 0.29 ± 0.27 | 0.007 | <0.001 | <0.001 |
|  | *Solobacterium* | % | 0.46 ± 0.34 | 0.60 ± 0.45 | 0.19 ± 0.22 | 0.018 | <0.001 | <0.001 |
|  | *Oribacterium* | % | 0.34 ± 0.21 | 0.42 ± 0.32 | 0.34 ± 0.31 | 0.115 | 1.000 | 0.003 |
|  | *Capnocytophaga* | % | 0.61 ± 0.78 | 0.14 ± 0.18 | 0.20 ± 0.23 | <0.001 | <0.001 | 0.303 |
|  | *Peptostreptococcus* | % | 0.40 ± 0.32 | 0.24 ± 0.30 | 0.09 ± 0.14 | <0.001 | <0.001 | <0.001 |
|  | *Eubacterium* | % | 0.22 ± 0.20 | 0.25 ± 0.23 | 0.05 ± 0.09 | 1.000 | <0.001 | <0.001 |
|  | *Parvimonas* | % | 0.31 ± 0.39 | 0.10 ± 0.19 | 0.14 ± 0.24 | <0.001 | <0.001 | 1.000 |
|  | *Corynebacterium* | % | 0.17 ± 0.79 | 0.14 ± 0.77 | 0.21 ± 0.59 | 1.000 | 0.168 | <0.001 |
|  | *Catonella* | % | 0.09 ± 0.10 | 0.08 ± 0.07 | 0.06 ± 0.08 | 1.000 | <0.001 | 0.014 |
|  | *Lautropia* | % | 0.11 ± 0.40 | 0.04 ± 0.06 | 0.08 ± 0.18 | <0.001 | 0.013 | 1.000 |
|  | *Treponema* | % | 0.06 ± 0.12 | 0.05 ± 0.11 | 0.11 ± 0.30 | 1.000 | 1.000 | 1.000 |
|  | *Olsenella* | % | 0.03 ± 0.07 | 0.06 ± 0.12 | 0.09 ± 0.22 | 0.135 | <0.001 | 1.000 |
|  | *Centipeda* | % | 0.03 ± 0.06 | 0.08 ± 0.10 | 0.03 ± 0.05 | <0.001 | 1.000 | <0.001 |
|  | *Aggregatibacter* | % | 0.07 ± 0.15 | 0.04 ± 0.06 | 0.05 ± 0.07 | <0.001 | 1.000 | 1.000 |
|  | *Abiotrophia* | % | 0.06 ± 0.10 | 0.03 ± 0.07 | 0.05 ± 0.10 | <0.001 | 0.007 | 1.000 |
|  | *Tannerella* | % | 0.03 ± 0.05 | 0.04 ± 0.08 | 0.05 ± 0.10 | 1.000 | 1.000 | 1.000 |
|  | *Selenomonas* | % | 0.03 ± 0.06 | 0.03 ± 0.04 | 0.04 ± 0.06 | 0.032 | 0.202 | 1.000 |
|  | *Butyrivibrio* | % | 0.02 ± 0.03 | 0.04 ± 0.07 | 0.01 ± 0.02 | <0.001 | <0.001 | <0.001 |
|  | *Dialister* | % | 0.02 ± 0.02 | 0.03 ± 0.05 | 0.03 ± 0.07 | <0.001 | 0.214 | 1.000 |
|  | *Peptococcus* | % | 0.03 ± 0.04 | 0.01 ± 0.03 | 0.01 ± 0.02 | <0.001 | <0.001 | 1.000 |
|  | *Cardiobacterium* | % | 0.02 ± 0.03 | 0.01 ± 0.01 | 0.02 ± 0.03 | 0.006 | 1.000 | <0.001 |
|  | *Slackia* | % | 0.01 ± 0.01 | 0.02 ± 0.03 | 0.02 ± 0.03 | <0.001 | 0.064 | 1.000 |
|  | *Kingella* | % | 0.01 ± 0.02 | 0.01 ± 0.02 | 0.02 ± 0.03 | <0.001 | <0.001 | 0.445 |
|  | *Schlegelella* | % | 0.01 ± 0.01 | 0.01 ± 0.01 | 0.02 ± 0.04 | 1.000 | <0.001 | <0.001 |
|  | *Mogibacterium* | % | 0.00 ± 0.01 | 0.01 ± 0.01 | 0.00 ± 0.01 | <0.001 | 1.000 | <0.001 |
|  | *Anaeroglobus* | % | 0.00 ± 0.01 | 0.01 ± 0.01 | 0.01 ± 0.03 | 0.630 | 0.270 | 1.000 |
|  | *Morococcus* | % | 0.01 ± 0.01 | 0.00 ± 0.00 | 0.00 ± 0.01 | <0.001 | <0.001 | 1.000 |
|  | Unclassified | % | 4.38 ± 2.21 | 2.78 ± 1.79 | 1.49 ± 1.15 | <0.001 | <0.001 | <0.001 |
| Species | *Streptococcus_Unclassified* | % | 14.23 ± 5.52 | 12.08 ± 6.10 | 20.80 ± 9.69 | <0.001 | <0.001 | <0.001 |
|  | *Streptococcus_salivarius* | % | 3.34 ± 3.18 | 9.95 ± 6.32 | 12.14 ± 9.25 | <0.001 | <0.001 | 1.000 |
|  | *Streptococcus_parasanguinis* | % | 1.08 ± 1.16 | 3.86 ± 2.81 | 6.51 ± 5.07 | <0.001 | <0.001 | <0.001 |
|  | *Streptococcus_sanguinis* | % | 0.12 ± 0.20 | 0.11 ± 0.12 | 0.32 ± 0.44 | 1.000 | <0.001 | <0.001 |
|  | *Streptococcus_gordonii* | % | 0.05 ± 0.12 | 0.11 ± 0.16 | 0.23 ± 0.36 | <0.001 | <0.001 | 0.475 |
|  | *Streptococcus_intermedius* | % | 0.01 ± 0.01 | 0.02 ± 0.03 | 0.04 ± 0.08 | 0.002 | <0.001 | 1.000 |
|  | *Prevotella_melaninogenica* | % | 7.43 ± 5.12 | 12.11 ± 7.40 | 5.29 ± 4.49 | <0.001 | 0.007 | <0.001 |
|  | *Prevotella_Unclassified* | % | 0.96 ± 1.58 | 5.69 ± 5.42 | 2.02 ± 3.17 | <0.001 | 0.003 | <0.001 |
|  | *Prevotella_pallens* | % | 1.17 ± 1.25 | 1.74 ± 1.61 | 0.26 ± 0.31 | <0.001 | <0.001 | <0.001 |
|  | *Prevotella_histicola* | % | 0.27 ± 0.75 | 1.92 ± 3.22 | 0.84 ± 1.78 | <0.001 | 0.152 | <0.001 |
|  | *Prevotella_vespertina* | % | 0.23 ± 0.54 | 1.31 ± 2.04 | 0.25 ± 0.50 | <0.001 | 1.000 | <0.001 |
|  | *Prevotella_aurantiaca* | % | 0.21 ± 0.49 | 0.05 ± 0.13 | 0.01 ± 0.11 | 1.000 | <0.001 | <0.001 |
|  | *Prevotella_nigrescens* | % | 0.03 ± 0.07 | 0.05 ± 0.11 | 0.06 ± 0.11 | 0.570 | 1.000 | 1.000 |
|  | *Prevotella_denticola* | % | 0.01 ± 0.04 | 0.03 ± 0.07 | 0.04 ± 0.09 | <0.001 | 0.004 | 1.000 |
|  | *Neisseria_Unclassified* | % | 19.71 ± 10.30 | 4.05 ± 4.60 | 4.77 ± 6.29 | <0.001 | <0.001 | 1.000 |
|  | *Neisseria_subflava* | % | 2.92 ± 7.64 | 0.79 ± 2.55 | 0.53 ± 2.09 | <0.001 | <0.001 | 1.000 |
|  | *Neisseria_flava* | % | 0.34 ± 0.55 | 0.23 ± 0.55 | 0.48 ± 1.12 | <0.001 | 1.000 | 1.000 |
|  | *Neisseria_elongata* | % | 0.11 ± 0.24 | 0.03 ± 0.06 | 0.06 ± 0.09 | <0.001 | 0.083 | 0.105 |
|  | *Abiotrophia_defectiva* | % | 0.07 ± 0.11 | 0.04 ± 0.09 | 0.06 ± 0.12 | <0.001 | 0.051 | 1.000 |
|  | *Actinomyces_graevenitzii* | % | 0.18 ± 0.22 | 0.45 ± 0.43 | 0.29 ± 0.41 | <0.001 | 1.000 | <0.001 |
|  | *Actinomyces_oris* | % | 0.02 ± 0.04 | 0.04 ± 0.07 | 0.11 ± 0.18 | <0.001 | <0.001 | <0.001 |
|  | *Actinomyces_Unclassified* | % | 0.01 ± 0.01 | 0.01 ± 0.02 | 0.02 ± 0.03 | 1.000 | 0.023 | 0.433 |
|  | *Aggregatibacter_Unclassified* | % | 0.07 ± 0.23 | 0.04 ± 0.08 | 0.05 ± 0.10 | 1.000 | 1.000 | 1.000 |
|  | *Alloprevotella_rava* | % | 0.11 ± 0.24 | 0.30 ± 0.71 | 0.04 ± 0.09 | 0.162 | 1.000 | <0.001 |
|  | *Alloprevotella_sp_HMT_308* | % | 0.68 ± 0.91 | 1.29 ± 1.34 | 0.34 ± 0.61 | <0.001 | 0.054 | <0.001 |
|  | *Alloprevotella_sp_HMT_914* | % | 1.50 ± 1.81 | 0.32 ± 0.80 | 0.13 ± 0.45 | <0.001 | <0.001 | 0.007 |
|  | *Alloprevotella_tannerae* | % | 0.13 ± 0.26 | 0.09 ± 0.21 | 0.04 ± 0.08 | 1.000 | 0.001 | 0.630 |
|  | *Alloprevotella_Unclassified* | % | 0.91 ± 2.15 | 0.18 ± 0.46 | 0.07 ± 0.21 | <0.001 | <0.001 | 0.020 |
|  | *Anaerovoracaceae_G1__sulci* | % | 0.32 ± 0.26 | 0.36 ± 0.32 | 0.07 ± 0.11 | 1.000 | <0.001 | <0.001 |
|  | *Butyrivibrio_sp_HMT_455* | % | 0.04 ± 0.07 | 0.09 ± 0.14 | 0.01 ± 0.03 | <0.001 | <0.001 | <0.001 |
|  | *Campylobacter_concisus* | % | 0.47 ± 0.47 | 0.78 ± 0.63 | 0.37 ± 0.34 | <0.001 | 1.000 | <0.001 |
|  | *Campylobacter_gracilis* | % | 0.01 ± 0.02 | 0.02 ± 0.03 | 0.03 ± 0.05 | 0.007 | <0.001 | 1.000 |
|  | *Campylobacter_showae* | % | 0.08 ± 0.17 | 0.04 ± 0.14 | 0.05 ± 0.11 | <0.001 | 0.110 | 1.000 |
|  | *Capnocytophaga_leadbetteri* | % | 0.24 ± 0.49 | 0.04 ± 0.08 | 0.06 ± 0.13 | <0.001 | <0.001 | 1.000 |
|  | *Capnocytophaga_ochracea* | % | 0.01 ± 0.02 | 0.02 ± 0.03 | 0.03 ± 0.05 | 1.000 | 0.017 | 1.000 |
|  | *Capnocytophaga_sputigena* | % | 0.16 ± 0.31 | 0.03 ± 0.05 | 0.04 ± 0.06 | <0.001 | <0.001 | 1.000 |
|  | *Capnocytophaga_Unclassified* | % | 0.23 ± 0.39 | 0.06 ± 0.11 | 0.06 ± 0.09 | <0.001 | <0.001 | 1.000 |
|  | *Cardiobacterium_hominis* | % | 0.01 ± 0.02 | 0.01 ± 0.02 | 0.02 ± 0.02 | 1.000 | 1.000 | 0.053 |
|  | *Catonella_Unclassified* | % | 0.10 ± 0.11 | 0.09 ± 0.08 | 0.05 ± 0.05 | 1.000 | <0.001 | <0.001 |
|  | *Corynebacterium_durum* | % | 0.03 ± 0.08 | 0.03 ± 0.06 | 0.07 ± 0.17 | 1.000 | 1.000 | 0.819 |
|  | *Corynebacterium_matruchotii* | % | 0.01 ± 0.01 | 0.01 ± 0.01 | 0.03 ± 0.09 | 1.000 | <0.001 | <0.001 |
|  | *Dialister_invisus* | % | 0.01 ± 0.02 | 0.03 ± 0.06 | 0.03 ± 0.08 | <0.001 | 0.123 | 1.000 |
|  | *Dialister_pneumosintes* | % | 0.01 ± 0.01 | 0.01 ± 0.03 | 0.02 ± 0.03 | 0.283 | 1.000 | 1.000 |
|  | *Fusobacterium_periodonticum* | % | 2.46 ± 1.65 | 1.20 ± 1.18 | 0.71 ± 0.97 | <0.001 | <0.001 | <0.001 |
|  | *Fusobacterium_Unclassified* | % | 0.45 ± 0.47 | 0.34 ± 0.42 | 0.62 ± 0.90 | 0.200 | 1.000 | 0.391 |
|  | *Fusobacterium_vincentii* | % | 0.02 ± 0.03 | 0.03 ± 0.06 | 0.06 ± 0.13 | 1.000 | 0.091 | 1.000 |
|  | *Gallibacter_brachus* | % | 0.02 ± 0.03 | 0.02 ± 0.04 | 0.06 ± 0.15 | 1.000 | 0.022 | 1.000 |
|  | *Gemella_morbillorum* | % | 0.01 ± 0.02 | 0.01 ± 0.02 | 0.02 ± 0.05 | 0.464 | 1.000 | 1.000 |
|  | *Gemella_sanguinis* | % | 1.25 ± 0.81 | 0.82 ± 0.85 | 1.42 ± 1.16 | <0.001 | 1.000 | <0.001 |
|  | *Gemella_Unclassified* | % | 0.87 ± 1.08 | 0.69 ± 0.75 | 1.37 ± 1.69 | 1.000 | 1.000 | 0.117 |
|  | *Granulicatella_adiacens* | % | 2.36 ± 1.23 | 1.94 ± 1.22 | 3.57 ± 2.16 | <0.001 | <0.001 | <0.001 |
|  | *Granulicatella_elegans* | % | 0.18 ± 0.38 | 0.12 ± 0.30 | 0.15 ± 0.49 | 1.000 | 0.005 | 1.000 |
|  | *Haemophilus_haemolyticus* | % | 0.27 ± 0.51 | 0.17 ± 0.39 | 0.23 ± 0.70 | 0.011 | 0.062 | 1.000 |
|  | *Haemophilus_parainfluenzae* | % | 7.21 ± 4.18 | 3.65 ± 3.21 | 7.84 ± 7.37 | <0.001 | 1.000 | <0.001 |
|  | *Haemophilus_sp_HMT_036* | % | 0.25 ± 0.49 | 0.15 ± 0.26 | 0.40 ± 1.30 | 0.597 | 1.000 | 1.000 |
|  | *Haemophilus_sputorum* | % | 0.10 ± 0.23 | 0.05 ± 0.12 | 0.09 ± 0.26 | 0.003 | <0.001 | 1.000 |
|  | *Haemophilus_Unclassified* | % | 0.59 ± 1.48 | 0.18 ± 0.49 | 0.41 ± 1.05 | <0.001 | <0.001 | 1.000 |
|  | *Hoylesella_nanceiensis* | % | 2.41 ± 2.15 | 1.16 ± 1.68 | 0.50 ± 0.79 | <0.001 | <0.001 | <0.001 |
|  | *Kingella_oralis* | % | 0.01 ± 0.01 | 0.02 ± 0.03 | 0.02 ± 0.04 | <0.001 | <0.001 | 0.292 |
|  | *Lachnoanaerobaculum_sp_HMT_083* | % | 0.33 ± 0.26 | 0.18 ± 0.21 | 0.11 ± 0.19 | <0.001 | <0.001 | 0.003 |
|  | *Lachnoanaerobaculum_Unclassified* | % | 0.13 ± 0.15 | 0.45 ± 0.40 | 0.19 ± 0.22 | <0.001 | 1.000 | <0.001 |
|  | *Lachnospiraceae_G2__bacteriumHMT_096* | % | 0.09 ± 0.20 | 0.27 ± 0.57 | 0.08 ± 0.18 | <0.001 | 1.000 | <0.001 |
|  | *Lancefieldella_parvula* | % | 0.02 ± 0.06 | 0.07 ± 0.17 | 0.02 ± 0.04 | <0.001 | 1.000 | 0.103 |
|  | *Lancefieldella_Unclassified* | % | 0.21 ± 0.21 | 0.90 ± 0.88 | 0.43 ± 0.47 | <0.001 | <0.001 | <0.001 |
|  | *Lautropia_mirabilis* | % | 0.13 ± 0.37 | 0.05 ± 0.07 | 0.09 ± 0.19 | <0.001 | 0.026 | 1.000 |
|  | *Leptotrichia_hongkongensis* | % | 0.01 ± 0.02 | 0.02 ± 0.04 | 0.05 ± 0.08 | <0.001 | <0.001 | 0.004 |
|  | *Leptotrichia_sp_HMT_221* | % | 0.09 ± 0.20 | 0.24 ± 0.40 | 0.11 ± 0.28 | <0.001 | 1.000 | <0.001 |
|  | *Leptotrichia_sp_HMT_417* | % | 0.23 ± 0.42 | 0.68 ± 1.20 | 0.15 ± 0.38 | <0.001 | 1.000 | <0.001 |
|  | *Megasphaera_micronuciformis* | % | 0.30 ± 0.40 | 1.91 ± 1.68 | 0.56 ± 0.62 | <0.001 | <0.001 | <0.001 |
|  | *Mogibacterium_Unclassified* | % | 0.01 ± 0.01 | 0.01 ± 0.01 | 0.01 ± 0.01 | <0.001 | 1.000 | <0.001 |
|  | *Moryella_sp_HMT_097* | % | 0.42 ± 0.46 | 0.39 ± 0.55 | 0.23 ± 0.36 | 1.000 | <0.001 | 0.029 |
|  | *Nanogingivalis_sp_HMT_399* | % | 0.09 ± 0.18 | 0.17 ± 0.39 | 0.07 ± 0.24 | 1.000 | 0.012 | <0.001 |
|  | *Nanosynbacter_Unclassified* | % | 1.36 ± 1.59 | 1.78 ± 1.90 | 0.40 ± 0.56 | 1.000 | <0.001 | <0.001 |
|  | *Nanosyncoccus_sp_HMT_351* | % | 0.28 ± 0.35 | 0.35 ± 0.37 | 0.09 ± 0.14 | 1.000 | <0.001 | <0.001 |
|  | *Oribacterium_asaccharolyticum* | % | 0.05 ± 0.07 | 0.16 ± 0.12 | 0.08 ± 0.14 | <0.001 | 1.000 | <0.001 |
|  | *Oribacterium_sinus* | % | 0.23 ± 0.18 | 0.27 ± 0.28 | 0.27 ± 0.27 | 1.000 | 1.000 | 1.000 |
|  | *Oribacterium_Unclassified* | % | 0.10 ± 0.11 | 0.08 ± 0.11 | 0.03 ± 0.08 | 0.002 | <0.001 | <0.001 |
|  | *Paludibacteraceae_G1__bacteriumHMT_274* | % | 0.01 ± 0.03 | 0.02 ± 0.04 | 0.04 ± 0.07 | 1.000 | 0.995 | 1.000 |
|  | *Parvimonas_micra* | % | 0.37 ± 0.43 | 0.14 ± 0.22 | 0.18 ± 0.29 | <0.001 | <0.001 | 1.000 |
|  | *Peptococcus_sp_HMT_168* | % | 0.05 ± 0.06 | 0.02 ± 0.04 | 0.01 ± 0.02 | <0.001 | <0.001 | 1.000 |
|  | *Peptostreptococcus_stomatis* | % | 0.63 ± 0.51 | 0.39 ± 0.46 | 0.15 ± 0.21 | <0.001 | <0.001 | <0.001 |
|  | *Porphyromonas_endodontalis* | % | 0.11 ± 0.24 | 0.05 ± 0.12 | 0.11 ± 0.23 | 1.000 | 1.000 | 1.000 |
|  | *Porphyromonas_pasteri* | % | 4.79 ± 2.95 | 1.11 ± 1.76 | 0.74 ± 1.21 | <0.001 | <0.001 | 0.879 |
|  | *Pseudoleptotrichia_sp_HMT_215* | % | 0.43 ± 0.56 | 0.32 ± 0.50 | 0.12 ± 0.28 | <0.001 | <0.001 | <0.001 |
|  | *Pseudoleptotrichia_Unclassified* | % | 0.15 ± 0.36 | 0.04 ± 0.10 | 0.04 ± 0.17 | <0.001 | <0.001 | 1.000 |
|  | *Riemerella_sp_HMT_322* | % | 0.14 ± 0.14 | 0.04 ± 0.05 | 0.06 ± 0.06 | <0.001 | <0.001 | 1.000 |
|  | *Rothia_aeria* | % | 0.04 ± 0.07 | 0.03 ± 0.05 | 0.06 ± 0.10 | 0.046 | 1.000 | 0.423 |
|  | *Rothia_dentocariosa* | % | 0.08 ± 0.13 | 0.13 ± 0.23 | 0.50 ± 1.12 | 0.039 | <0.001 | <0.001 |
|  | *Rothia_mucilaginosa* | % | 1.28 ± 1.04 | 1.27 ± 0.96 | 4.68 ± 3.11 | 1.000 | <0.001 | <0.001 |
|  | *Ruminococcaceae_G1__bacteriumHMT_075* | % | 0.07 ± 0.12 | 0.05 ± 0.09 | 0.02 ± 0.03 | 0.198 | <0.001 | 0.445 |
|  | *Ruminococcaceae_G2__bacteriumHMT_085* | % | 0.11 ± 0.15 | 0.14 ± 0.15 | 0.04 ± 0.06 | 1.000 | <0.001 | <0.001 |
|  | *Saccharimonas_sp_HMT_346* | % | 0.01 ± 0.02 | 0.02 ± 0.03 | 0.03 ± 0.05 | 1.000 | 1.000 | 1.000 |
|  | *Schaalia_lingnae* | % | 0.07 ± 0.08 | 0.16 ± 0.18 | 0.24 ± 0.28 | <0.001 | <0.001 | 0.738 |
|  | *Schaalia_odontolytica* | % | 0.15 ± 0.17 | 0.18 ± 0.25 | 0.29 ± 0.46 | 1.000 | 1.000 | 1.000 |
|  | *Schaalia_Unclassified* | % | 0.64 ± 0.57 | 1.25 ± 1.16 | 0.75 ± 0.66 | <0.001 | 1.000 | <0.001 |
|  | *Segatella_oris* | % | 0.05 ± 0.07 | 0.09 ± 0.13 | 0.13 ± 0.20 | <0.001 | 0.008 | 1.000 |
|  | *Segatella_salivae* | % | 0.40 ± 0.47 | 1.73 ± 1.48 | 0.48 ± 0.53 | <0.001 | 1.000 | <0.001 |
|  | *Segatella_sp_HMT_305* | % | 0.02 ± 0.04 | 0.11 ± 0.14 | 0.03 ± 0.05 | <0.001 | 0.002 | <0.001 |
|  | *Selenomonas_artemidis* | % | 0.09 ± 0.13 | 0.19 ± 0.23 | 0.38 ± 0.58 | <0.001 | <0.001 | 0.644 |
|  | *Selenomonas_felix* | % | 0.05 ± 0.10 | 0.15 ± 0.18 | 0.05 ± 0.08 | <0.001 | 1.000 | <0.001 |
|  | *Selenomonas_noxia* | % | 0.01 ± 0.01 | 0.01 ± 0.02 | 0.02 ± 0.03 | 0.267 | 0.003 | 1.000 |
|  | *Selenomonas_sputigena* | % | 0.01 ± 0.02 | 0.01 ± 0.03 | 0.02 ± 0.04 | 0.012 | 1.000 | 1.000 |
|  | *Selenomonas_Unclassified* | % | 0.02 ± 0.06 | 0.03 ± 0.05 | 0.03 ± 0.04 | 1.000 | 1.000 | 1.000 |
|  | *Solobacterium_moorei* | % | 0.30 ± 0.22 | 0.42 ± 0.32 | 0.14 ± 0.15 | 0.003 | <0.001 | <0.001 |
|  | *Stomatobaculum_Unclassified* | % | 0.03 ± 0.08 | 0.35 ± 0.49 | 0.17 ± 0.28 | <0.001 | <0.001 | <0.001 |
|  | *Tannerella_forsythia* | % | 0.01 ± 0.02 | 0.02 ± 0.04 | 0.03 ± 0.07 | 1.000 | 0.054 | 1.000 |
|  | *Treponema_Unclassified* | % | 0.03 ± 0.05 | 0.03 ± 0.06 | 0.04 ± 0.11 | 1.000 | 1.000 | 1.000 |
|  | *Veillonella_atypica* | % | 0.76 ± 0.96 | 4.25 ± 3.14 | 2.38 ± 2.36 | <0.001 | <0.001 | <0.001 |
|  | *Veillonella_parvula* | % | 0.03 ± 0.04 | 0.06 ± 0.11 | 0.09 ± 0.16 | 0.006 | <0.001 | 1.000 |
|  | *Veillonella_rogosae* | % | 2.11 ± 1.57 | 0.84 ± 1.40 | 1.20 ± 1.96 | <0.001 | <0.001 | 1.000 |
|  | *Veillonella_Unclassified* | % | 1.62 ± 1.25 | 1.01 ± 1.12 | 1.20 ± 1.38 | <0.001 | 0.002 | 1.000 |
|  | *Unclassified_Unclassified* | % | 1.76 ± 1.85 | 6.15 ± 3.19 | 5.99 ± 4.66 | <0.001 | <0.001 | 1.000 |
|  | OtherGenera | % | 3.42 ± 3.25 | 2.15 ± 2.24 | 2.39 ± 3.69 | <0.001 | <0.001 | 1.000 |

Each variable is expressed as mean ± standard deviation. p value was calculated by the Wilcoxon rank sum test with Bonferroni correction.

Supplementary Table 2: Characteristics by orotype

| Category | Variabels | Unit or Level | Overall | N type | P type | S type | *p* |
| --- | --- | --- | --- | --- | --- | --- | --- |
|  | n |  | 644 | 237 | 286 | 121 |  |
| Demographic information | Age |  | 53.4±14.7 | 52.1±14.4 | 53.4±14.7 | 55.8±14.8 | 0.07 |
|  | Sex | Male | 43.0 (277) | 39.2 ( 93) | 41.6 (119) | 53.7 ( 65) | 0.026 |
|  |  | Female | 57.0 (367) | 60.8 (144) | 58.4 (167) | 46.3 ( 56) |  |
|  | Educational background | Primary school | 0.2 (  1) | 0.0 (  0) | 0.0 (  0) | 0.8 (  1) | 0.02 |
|  |  | Junior high school | 6.4 ( 41) | 5.5 ( 13) | 5.2 ( 15) | 10.7 ( 13) |  |
|  |  | High school | 56.7 (365) | 51.9 (123) | 59.8 (171) | 58.7 ( 71) |  |
|  |  | Technical school | 24.7 (159) | 27.0 ( 64) | 25.9 ( 74) | 17.4 ( 21) |  |
|  |  | College | 12.1 ( 78) | 15.6 ( 37) | 9.1 ( 26) | 12.4 ( 15) |  |
|  | Cohabits number |  | 3.7±1.6 | 3.7±1.6 | 3.9±1.5 | 3.5±1.6 | 0.023 |
|  | Spouse | No | 13.8 ( 89) | 15.6 ( 37) | 10.8 ( 31) | 17.4 ( 21) | 0.003 |
|  |  | Bereavement | 3.9 ( 25) | 5.1 ( 12) | 2.4 (  7) | 5.0 (  6) |  |
|  |  | Divorce | 5.1 ( 33) | 8.9 ( 21) | 3.1 (  9) | 2.5 (  3) |  |
|  |  | Yes | 77.2 (497) | 70.5 (167) | 83.6 (239) | 75.2 ( 91) |  |
| Lifestyle | Smoking | Never | 50.2 (323) | 56.1 (133) | 47.2 (135) | 45.5 ( 55) | 0.053 |
|  |  | Current | 16.9 (109) | 11.8 ( 28) | 20.6 ( 59) | 18.2 ( 22) |  |
|  |  | Past | 32.9 (212) | 32.1 ( 76) | 32.2 ( 92) | 36.4 ( 44) |  |
|  | Passive smoking | No | 65.8 (424) | 70.0 (166) | 63.6 (182) | 62.8 ( 76) | 0.226 |
|  |  | Yes | 34.2 (220) | 30.0 ( 71) | 36.4 (104) | 37.2 ( 45) |  |
|  | Drinking | Never | 42.5 (274) | 41.8 ( 99) | 45.1 (129) | 38.0 ( 46) | 0.34 |
|  |  | Current | 51.9 (334) | 54.4 (129) | 48.3 (138) | 55.4 ( 67) |  |
|  |  | Past | 5.6 ( 36) | 3.8 (  9) | 6.6 ( 19) | 6.6 (  8) |  |
|  | Alcoholic beverage | g/day | 230.6±399 | 208.3±355.5 | 218.6±358.5 | 302.6±541 | 0.084 |
|  | Exercise | Everyday | 24.5 (158) | 21.9 ( 52) | 26.6 ( 76) | 24.8 ( 30) | 0.575 |
|  |  | 5-6/week | 17.9 (115) | 17.7 ( 42) | 19.9 ( 57) | 13.2 ( 16) |  |
|  |  | 2-4/week | 22.5 (145) | 24.1 ( 57) | 20.6 ( 59) | 24.0 ( 29) |  |
|  |  | 1 or less/week | 12.4 ( 80) | 12.2 ( 29) | 10.8 ( 31) | 16.5 ( 20) |  |
|  |  | No | 22.7 (146) | 24.1 ( 57) | 22.0 ( 63) | 21.5 ( 26) |  |
|  | Sports | No | 73.3 (472) | 68.8 (163) | 76.9 (220) | 73.6 ( 89) | 0.111 |
|  |  | Yes | 26.7 (172) | 31.2 ( 74) | 23.1 ( 66) | 26.4 ( 32) |  |
|  | Sleep (JESS) |  | 6.2±4.2 | 5.9±4.1 | 6.5±4.4 | 6±4 | 0.287 |
| Dietary habits | Grains | g/day | 466.1±147.6 | 452.3±161.5 | 476.7±136.9 | 467.9±142.5 | 0.166 |
|  | Potato | g/day | 27±24.3 | 27.8±23.1 | 26.7±23.9 | 26.3±27.6 | 0.822 |
|  | Sugar and sweeteners | g/day | 0.22±1.31 | 0.07±0.55 | 0.37±1.8 | 0.17±0.93 | 0.035 |
|  | Pulse | g/day | 82.5±120.4 | 94.8±139 | 77.1±113.1 | 71.1±93.9 | 0.128 |
|  | Nuts and bolts | g/day | 2.11±4.56 | 1.94±3.58 | 2.2±5.35 | 2.22±4.24 | 0.775 |
|  | Vegetables | g/day | 219.8±162.9 | 244.8±171.2 | 210.6±169.4 | 192.6±119.5 | 0.007 |
|  | Green and yellow vegetables | g/day | 98.9±92 | 113.8±102.5 | 90.2±89.3 | 90.2±71.2 | 0.007 |
|  | Other vegetables | g/day | 120.9±101.3 | 130.9±103.4 | 120.4±107.7 | 102.5±76.9 | 0.042 |
|  | Fruits | g/day | 142.9±155.1 | 130.3±143.8 | 161.4±173.2 | 123.9±124.7 | 0.024 |
|  | Mushrooms | g/day | 10.5±10.9 | 11.5±11.1 | 10.7±10.5 | 8.2±11 | 0.022 |
|  | Seaweed | g/day | 7.26±9.69 | 8.03±13.57 | 7.09±6.56 | 6.14±6.03 | 0.201 |
|  | Seafood | g/day | 50.6±38.2 | 51±40.4 | 49.5±34.1 | 52.5±42.7 | 0.753 |
|  | Meats | g/day | 81.4±55.4 | 87.2±60.4 | 78.8±53.4 | 76.4±49 | 0.123 |
|  | Eggs | g/day | 42.2±58.9 | 50.2±70.3 | 34.7±39.5 | 44.4±70.1 | 0.01 |
|  | Dairy | g/day | 277.5±369.6 | 307.9±401.6 | 265.3±368.6 | 246.6±298.3 | 0.252 |
|  | Oils and fats | g/day | 12.3±7.8 | 12.4±6.5 | 12.4±7.8 | 11.7±9.7 | 0.717 |
|  | Snacks | g/day | 25.4±26 | 22.3±19.8 | 28.1±25.1 | 25.2±36.4 | 0.038 |
|  | Discretionary beverages | g/day | 643.8±475.6 | 602±471.7 | 638.1±449.6 | 739.2±530.9 | 0.034 |
|  | Seasoning and spices | g/day | 22.3±12.7 | 23.1±12.5 | 22.1±12.2 | 21.1±14.4 | 0.349 |
| Oral care | Brushing | Number/day | 2.2±0.8 | 2.4±0.9 | 2.2±0.8 | 2±0.8 | <0.001 |
|  | Dental clinic (treatment) | No | 70.3 (453) | 73.0 (173) | 66.1 (189) | 75.2 ( 91) | 0.097 |
|  |  | Yes | 29.7 (191) | 27.0 ( 64) | 33.9 ( 97) | 24.8 ( 30) |  |
|  | Dental clinic (checkup or cleaning) | No | 67.5 (435) | 63.3 (150) | 68.5 (196) | 73.6 ( 89) | 0.13 |
|  |  | Yes | 32.5 (209) | 36.7 ( 87) | 31.5 ( 90) | 26.4 ( 32) |  |
| Medical history | Hypertension | Never | 74.1 (477) | 77.6 (184) | 74.8 (214) | 65.3 ( 79) | 0.058 |
|  |  | Current | 24.1 (155) | 20.7 ( 49) | 22.7 ( 65) | 33.9 ( 41) |  |
|  |  | Past | 1.9 ( 12) | 1.7 (  4) | 2.4 (  7) | 0.8 (  1) |  |
|  | Diabetes | Never | 92.1 (593) | 94.5 (224) | 91.6 (262) | 88.4 (107) | 0.32 |
|  |  | Current | 6.8 ( 44) | 5.1 ( 12) | 7.0 ( 20) | 9.9 ( 12) |  |
|  |  | Past | 1.1 (  7) | 0.4 (  1) | 1.4 (  4) | 1.7 (  2) |  |
|  | Hyperlipidemia | Never | 82.0 (528) | 85.2 (202) | 82.9 (237) | 73.6 ( 89) | 0.059 |
|  |  | Current | 12.9 ( 83) | 10.5 ( 25) | 11.5 ( 33) | 20.7 ( 25) |  |
|  |  | Past | 5.1 ( 33) | 4.2 ( 10) | 5.6 ( 16) | 5.8 (  7) |  |
|  | Osteoporosis | Never | 96.4 (621) | 96.2 (228) | 95.8 (274) | 98.3 (119) | 0.613 |
|  |  | Current | 3.0 ( 19) | 3.4 (  8) | 3.1 (  9) | 1.7 (  2) |  |
|  |  | Past | 0.6 (  4) | 0.4 (  1) | 1.0 (  3) | 0.0 (  0) |  |
|  | Bronchial asthma | Never | 91.0 (586) | 89.9 (213) | 92.0 (263) | 90.9 (110) | 0.949 |
|  |  | Current | 3.1 ( 20) | 3.4 (  8) | 2.8 (  8) | 3.3 (  4) |  |
|  |  | Past | 5.9 ( 38) | 6.8 ( 16) | 5.2 ( 15) | 5.8 (  7) |  |
|  | Depression | Never | 96.3 (620) | 97.0 (230) | 95.5 (273) | 96.7 (117) | 0.885 |
|  |  | Current | 2.2 ( 14) | 1.7 (  4) | 2.8 (  8) | 1.7 (  2) |  |
|  |  | Past | 1.6 ( 10) | 1.3 (  3) | 1.7 (  5) | 1.7 (  2) |  |
|  | Dementia | Never | 99.7 (642) | 100.0 (237) | 99.7 (285) | 99.2 (120) | 0.233 |
|  |  | Current | 0.2 (  1) | 0.0 (  0) | 0.3 (  1) | 0.0 (  0) |  |
|  |  | Past | 0.2 (  1) | 0.0 (  0) | 0.0 (  0) | 0.8 (  1) |  |

Each numerical variable is expressed as mean ± standard deviation, and each categorical variable is expressed as a percentage (N).

p‑value was calculated by the Kruskal-Wallis test (numerical variables) and Fisher’s exact test (categorical variables). Red numbers represent p<0.05.

Supplementary Table 3: Result of variable selection

| Attributes and lifestyle |  | Diet |  | Dental questionnaire |  |
| --- | --- | --- | --- | --- | --- |
| Age | 〇 | Grains |  | Brushing | ● |
| Sex | ● | Potato |  | Dental clinic (treatment) | 〇 |
| Educational background | ● | Sugar and sweeteners | ● | Dental clinic (cleaning or checkup) |  |
| Cohabits number | ● | Pulse |  |  |  |
| Spouse | ● | Nuts and bolts |  |  |  |
| Smoking | 〇 | Vegetables | ● |  |  |
| Passive smoking |  | Fruits | ● |  |  |
| Drinking |  | Mushrooms | ● |  |  |
| Alcoholic beverage | 〇 | Seaweed |  |  |  |
| Exercise |  | Seafood |  |  |  |
| Sports |  | Meats |  |  |  |
| Sleep |  | Eggs | ● |  |  |
|  |  | Dairy |  |  |  |
|  |  | Oils and fats |  |  |  |
|  |  | Snacks | ● |  |  |
|  |  | Discretionary beverages | ● |  |  |
|  |  | Seasoning and spices |  |  |  |

●: p < 0.05, 〇: p < 0.1

Supplementary Table 4: Associations between orotypes and lifestyle factors by logistic regression analysis

|  |  | N type (Ref: Rest) | | | | P type (Ref: Rest) | | | | S type (Ref: Rest) | | | |
| --- | --- | --- | --- | --- | --- | --- | --- | --- | --- | --- | --- | --- | --- |
| Exposure | Level | OR | Lower | Upper | P | OR | Lower | Upper | P | OR | Lower | Upper | P |
| Sex | Male (Ref: Female) | 1.14 | 0.74 | 1.77 | 0.556 | 0.79 | 0.51 | 1.20 | 0.266 | 1.16 | 0.69 | 1.95 | 0.573 |
| Age | Every 10 years | 0.90 | 0.79 | 1.03 | 0.130 | 1.03 | 0.90 | 1.17 | 0.653 | 1.10 | 0.94 | 1.30 | 0.242 |
| Educational Background | Numeric^a^ | 1.11 | 0.73 | 1.70 | 0.623 | 1.01 | 0.67 | 1.53 | 0.961 | 0.85 | 0.51 | 1.42 | 0.531 |
| Cohabits | Number | 0.97 | 0.86 | 1.09 | 0.582 | 1.11 | 1.00 | 1.25 | **0.061** | 0.89 | 0.77 | 1.03 | 0.112 |
| Spouse | Yes (Ref: No) | 0.51 | 0.33 | 0.77 | 0.002 | 2.12 | 1.38 | 3.26 | 0.001 | 0.97 | 0.58 | 1.63 | 0.914 |
| Smoking | Current (Ref: Never) | 0.57 | 0.33 | 0.99 | 0.048 | 1.97 | 1.18 | 3.31 | 0.010 | 0.79 | 0.42 | 1.49 | 0.473 |
|  | Past (Ref: Never) | 0.88 | 0.58 | 1.35 | 0.571 | 1.19 | 0.79 | 1.80 | 0.410 | 0.94 | 0.56 | 1.57 | 0.814 |
| Alcohol | Every 1SD | 0.94 | 0.77 | 1.16 | 0.562 | 1.03 | 0.85 | 1.26 | 0.736 | 1.07 | 0.86 | 1.34 | 0.526 |
| Sugar and Sweeteners | Every 1SD | 0.75 | 0.59 | 0.95 | 0.018 | 1.33 | 1.09 | 1.62 | 0.005 | 0.93 | 0.73 | 1.17 | 0.522 |
| Vegetables | Every 1SD | 1.54 | 1.20 | 1.97 | 0.001 | 0.69 | 0.54 | 0.87 | 0.002 | 1.01 | 0.79 | 1.31 | 0.912 |
| Fruits | Every 1SD | 0.71 | 0.57 | 0.89 | 0.003 | 1.36 | 1.10 | 1.66 | 0.004 | 1.00 | 0.79 | 1.27 | 0.992 |
| Mushrooms | Every 1SD | 1.12 | 0.92 | 1.37 | 0.260 | 1.10 | 0.90 | 1.34 | 0.349 | 0.72 | 0.56 | 0.93 | 0.010 |
| Eggs | Every 1SD | 1.14 | 0.94 | 1.39 | 0.176 | 0.88 | 0.74 | 1.06 | 0.170 | 1.04 | 0.84 | 1.28 | 0.716 |
| Snacks | Every 1SD | 0.78 | 0.64 | 0.96 | 0.019 | 1.33 | 1.09 | 1.64 | 0.006 | 0.99 | 0.79 | 1.25 | 0.941 |
| Discretionary Beverages | Every 1SD | 0.98 | 0.82 | 1.18 | 0.854 | 0.87 | 0.73 | 1.05 | 0.139 | 1.34 | 1.05 | 1.72 | 0.020 |
| Brushing | Number | 1.30 | 1.04 | 1.61 | 0.020 | 0.95 | 0.77 | 1.18 | 0.634 | 0.73 | 0.55 | 0.97 | 0.028 |
| Dental Clinic (Treatment) | Yes (Ref: No) | 0.82 | 0.56 | 1.19 | 0.295 | 1.39 | 0.97 | 2.00 | **0.071** | 0.79 | 0.49 | 1.26 | 0.319 |

a: Converted to the following numeric types for analysis. 1: Primary school or junior high school, 2: High school, 3: Technical school, 4: College

OR, odds ratio. Red numbers represent p<0.05.

Supplementary Table 5: Health status by orotype

| Category | Variables | Unit or Level | Overall | N type | P type | S type | *p* |
| --- | --- | --- | --- | --- | --- | --- | --- |
|  | n |  | 644 | 237 | 286 | 121 |  |
| Oral | Teeth | Number | 24.7±6.6 | 26.1±5.5 | 24.3±6.6 | 22.8±8.2 | <0.001 |
|  | Eichner | A1 | 43.5 (280) | 50.2 (119) | 41.6 (119) | 34.7 ( 42) | 0.015 |
|  |  | A2 | 21.1 (136) | 22.4 ( 53) | 21.7 ( 62) | 17.4 ( 21) |  |
|  |  | A3 | 5.7 ( 37) | 5.5 ( 13) | 6.3 ( 18) | 5.0 (  6) |  |
|  |  | B1 | 10.2 ( 66) | 9.3 ( 22) | 10.5 ( 30) | 11.6 ( 14) |  |
|  |  | B2 | 5.9 ( 38) | 5.9 ( 14) | 5.2 ( 15) | 7.4 (  9) |  |
|  |  | B3 | 4.0 ( 26) | 1.3 (  3) | 4.9 ( 14) | 7.4 (  9) |  |
|  |  | B4 | 3.1 ( 20) | 2.5 (  6) | 3.8 ( 11) | 2.5 (  3) |  |
|  |  | C1 | 1.2 (  8) | 0.4 (  1) | 1.4 (  4) | 2.5 (  3) |  |
|  |  | C2 | 2.3 ( 15) | 1.7 (  4) | 1.7 (  5) | 5.0 (  6) |  |
|  |  | C3 | 2.8 ( 18) | 0.8 (  2) | 2.8 (  8) | 6.6 (  8) |  |
|  | Total caries | Number | 1±2.3 | 0.7±1.7 | 0.9±2.1 | 1.6±3.5 | 0.005 |
|  | Mild caries | Number | 0.7±1.6 | 0.6±1.6 | 0.7±1.6 | 0.9±1.6 | 0.376 |
|  | Severe caries | Number | 0.3±1.3 | 0.1±0.5 | 0.2±0.9 | 0.7±2.6 | <0.001 |
|  | Periodontal disease | < 4mm | 45.0 (290) | 50.6 (120) | 44.1 (126) | 36.4 ( 44) | 0.144 |
|  |  | < 6mm | 44.6 (287) | 39.7 ( 94) | 45.5 (130) | 52.1 ( 63) |  |
|  |  | ≥ 6mm | 10.4 ( 67) | 9.7 ( 23) | 10.5 ( 30) | 11.6 ( 14) |  |
|  | OHIP14 Total |  | 4.8±6.9 | 3.8±6 | 5±7 | 6.6±8.1 | 0.001 |
|  | OHIP14 SD1 (Functional limitaion) |  | 0.7±1.3 | 0.6±1.2 | 0.7±1.4 | 0.9±1.4 | 0.173 |
|  | OHIP14 SD2 (Physical pain) |  | 0.9±1.5 | 0.8±1.4 | 0.9±1.5 | 1.1±1.6 | 0.159 |
|  | OHIP14 SD3 (Psychological discomfort) |  | 0.8±1.3 | 0.5±1 | 0.8±1.3 | 1.1±1.6 | <0.001 |
|  | OHIP14 SD4 (Physical disability) |  | 0.5±1 | 0.5±1 | 0.5±1 | 0.7±1.2 | 0.07 |
|  | OHIP14 SD5 (Psycological disability) |  | 0.7±1.2 | 0.5±1 | 0.7±1.2 | 1±1.5 | 0.002 |
|  | OHIP14 SD6 (Social disability) |  | 0.6±1.1 | 0.5±1 | 0.6±1.1 | 0.9±1.4 | 0.001 |
|  | OHIP14 SD7 (Handicap) |  | 0.6±1.1 | 0.4±0.9 | 0.7±1.2 | 0.9±1.3 | 0.001 |
|  | Salivary IgA | µg/mL | 135.1±308.1 | 111.2±309 | 131.4±224.5 | 190.7±443.9 | 0.067 |
|  | Salivation | mL/min | 0.72±0.57 | 0.75±0.58 | 0.75±0.59 | 0.61±0.46 | 0.056 |
| MetS | Abdominal circumference | cm | 83.6±9.9 | 82.1±9.5 | 83.5±9.2 | 86.7±11.4 | <0.001 |
|  | Systole blood pressure | mmHg | 129.9±19.7 | 128.1±19.7 | 129.5±19.2 | 134.6±20.4 | 0.01 |
|  | Diastole blood pressure | mmHg | 82±11.4 | 81.5±11.4 | 81.4±11.1 | 84.5±12 | 0.033 |
|  | Fasting blood glucose | mg/dL | 100.7±22.1 | 97.6±14.6 | 101.2±22.7 | 105.5±30.6 | 0.005 |
|  | HDL cholesterol | mg/dL | 70.8±18.4 | 73.7±18.1 | 69±18 | 69.4±19.5 | 0.01 |
|  | Triglycerides | mg/dL | 107.3±101.4 | 100.8±112.5 | 106±84.2 | 123±114.2 | 0.141 |
|  | HbA1c | % | 5.7±0.8 | 5.6±0.4 | 5.7±0.9 | 5.9±1.1 | 0.002 |
|  | Total cholesterol | mg/dL | 216.6±36.7 | 216.8±35.5 | 217.4±38.8 | 214.2±34.1 | 0.725 |
|  | LDL cholesterol | mg/dL | 123.5±31.4 | 122.3±32.1 | 125.8±31.3 | 120.3±30 | 0.215 |
|  | Ankle Brachial Index |  | 1.1±0.1 | 1.1±0.1 | 1.1±0.1 | 1.1±0.1 | 0.11 |
|  | Cardio-Ankle Vascular Index |  | 7.5±1.3 | 7.3±1.3 | 7.4±1.2 | 7.8±1.3 | 0.001 |
| Liver | AST | U/L | 22.7±8.4 | 22±6.5 | 22.2±8.5 | 25.4±10.7 | <0.001 |
|  | ALT | U/L | 21.7±14.3 | 19.9±10.7 | 20.6±11.1 | 27.6±23.1 | <0.001 |
|  | GGT | U/L | 35.4±39.8 | 33.5±36.9 | 32.8±37.6 | 45.4±48.3 | 0.009 |
| Inflammation | High Sensitive C-reactive Protein | mg/dL | 0.1±0.3 | 0.1±0.2 | 0.1±0.2 | 0.1±0.4 | 0.482 |
|  | IL-6 | pg/mL | 2.1±5.1 | 2.5±7.7 | 1.7±2.4 | 2.1±3.0 | 0.215 |
|  | TNF-α | pg/mL | 5.6±2.8 | 5.5±3.4 | 5.5±2.5 | 5.8±2 | 0.63 |

Each numerical variable is expressed as mean ± standard deviation, and each categorical variable is expressed as a percentage (N).

p‑value was calculated by the Kruskal-Wallis test (numerical variables) and Fisher’s exact test (categorical variables). Red numbers represent p<0.05.

Supplementary Table 6: Associations between orotypes and health status by logistic regression analysis and multiple regression analysis

|  |  |  |  | **Logistic regression using binominal data by cutoff value** | | | | | | | | | **Linear regression using numeric data** | | | | | | | | |
| --- | --- | --- | --- | --- | --- | --- | --- | --- | --- | --- | --- | --- | --- | --- | --- | --- | --- | --- | --- | --- | --- |
|  |  |  |  | N type | P type | | | | S type | | | | N type | P type | | | | S type | | | |
|  |  | Unit | Cutoff | OR | OR | Lower | Upper | P | OR | Lower | Upper | P | β | β | Lower | Upper | P | β | Lower | Upper | P |
| Oral | Teeth | Number | < 20 | Ref | 1.36 | 0.67 | 2.80 | 0.395 | **2.50** | **1.11** | **5.63** | **0.027** | Ref | **-0.95** | **-1.90** | **0.00** | **0.050** | **-1.69** | **-2.89** | **-0.49** | **0.006** |
|  | Eichner |  | B1 – C4 | Ref | 1.35 | 0.82 | 2.20 | 0.237 | **2.08** | **1.16** | **3.73** | **0.014** |  |  |  |  |  |  |  |  |  |
|  | Periodontal disease |  | PPD ≥ 4 mm | Ref | 1.26 | 0.87 | 1.82 | 0.218 | **1.66** | **1.04** | **2.68** | **0.035** |  |  |  |  |  |  |  |  |  |
|  | Caries | Number | ≥ 1 | Ref | **1.46** | **0.98** | **2.18** | **0.061** | 1.48 | 0.90 | 2.42 | 0.120 | Ref | 0.07 | -0.34 | 0.48 | 0.742 | **0.64** | **0.12** | **1.15** | **0.015** |
|  | OHIP Total |  | ≥ 1 | Ref | 1.11 | 0.76 | 1.61 | 0.597 | **1.78** | **1.11** | **2.88** | **0.017** | Ref | 0.74 | -0.44 | 1.92 | 0.220 | **2.20** | **0.72** | **3.69** | **0.004** |
|  | OHIP SD1 |  | ≥ 1 | Ref | 0.95 | 0.61 | 1.48 | 0.829 | 1.20 | 0.70 | 2.04 | 0.509 | Ref | 0.04 | -0.18 | 0.26 | 0.700 | 0.12 | -0.15 | 0.40 | 0.384 |
|  | OHIP SD2 |  | ≥ 1 | Ref | 1.06 | 0.70 | 1.59 | 0.797 | 1.25 | 0.76 | 2.06 | 0.387 | Ref | 0.05 | -0.21 | 0.30 | 0.717 | 0.23 | -0.09 | 0.55 | 0.164 |
|  | OHIP SD3 |  | ≥ 1 | Ref | **1.42** | **0.95** | **2.14** | **0.088** | **1.60** | **0.97** | **2.63** | **0.067** | Ref | **0.21** | **-0.01** | **0.43** | **0.065** | **0.48** | **0.20** | **0.76** | **0.001** |
|  | OHIP SD4 |  | ≥ 1 | Ref | 1.21 | 0.77 | 1.89 | 0.415 | 1.49 | 0.87 | 2.56 | 0.145 | Ref | 0.02 | -0.16 | 0.20 | 0.858 | **0.20** | **-0.03** | **0.42** | **0.092** |
|  | OHIP SD5 |  | ≥ 1 | Ref | **1.42** | **0.94** | **2.16** | **0.094** | **1.69** | **1.02** | **2.80** | **0.041** | Ref | **0.19** | **-0.02** | **0.40** | **0.070** | **0.42** | **0.16** | **0.68** | **0.002** |
|  | OHIP SD6 |  | ≥ 1 | Ref | 1.41 | 0.90 | 2.20 | 0.131 | **2.26** | **1.33** | **3.84** | **0.003** | Ref | 0.09 | -0.10 | 0.28 | 0.368 | **0.39** | **0.15** | **0.63** | **0.002** |
|  | OHIP SD7 |  | ≥ 1 | Ref | 1.28 | 0.83 | 1.99 | 0.267 | **1.98** | **1.17** | **3.34** | **0.011** | Ref | 0.15 | -0.05 | 0.34 | 0.135 | **0.37** | **0.13** | **0.61** | **0.003** |
|  | Salivary IgA | µg/mL |  |  |  |  |  |  |  |  |  |  | Ref | 11.68 | -43.17 | 66.53 | 0.676 | **68.59** | **-0.47** | **137.65** | **0.052** |
|  | Salivation | mL/min |  |  |  |  |  |  |  |  |  |  | Ref | -0.04 | -0.14 | 0.06 | 0.476 | **-0.15** | **-0.27** | **-0.02** | **0.022** |
| MetS | Abdominal circumference | cm | ≥ 85 (Male) ≥ 90 (Female) | Ref | 0.96 | 0.61 | 1.52 | 0.875 | **2.21** | **1.26** | **3.86** | **0.005** | Ref | 0.59 | -1.00 | 2.17 | 0.469 | **2.60** | **0.60** | **4.60** | **0.011** |
|  | Systole blood pressure | mmHg | ≥ 130 | Ref | 1.16 | 0.75 | 1.81 | 0.501 | 1.46 | 0.84 | 2.54 | 0.177 | Ref | 0.68 | -2.18 | 3.54 | 0.642 | 2.73 | -0.88 | 6.33 | 0.138 |
|  | Diastole blood pressure | mmHg | ≥ 85 | Ref | 1.37 | 0.92 | 2.05 | 0.122 | **1.53** | **0.93** | **2.51** | **0.092** | Ref | -0.01 | -1.93 | 1.91 | 0.992 | 1.76 | -0.67 | 4.18 | 0.156 |
|  | Fasting blood glucose | mg/dL | ≥ 110 | Ref | 1.39 | 0.79 | 2.42 | 0.250 | 1.54 | 0.81 | 2.94 | 0.187 | Ref | 1.52 | -2.20 | 5.25 | 0.423 | **4.24** | **-0.45** | **8.93** | **0.077** |
|  | HDL cholesterol | mg/dL | < 40 | Ref | 2.61 | 0.48 | 14.21 | 0.267 | 2.39 | 0.34 | 16.82 | 0.383 | Ref | **-3.84** | **-6.98** | **-0.69** | **0.017** | -2.34 | -6.30 | 1.62 | 0.248 |
|  | Triglycerides | mg/dL | ≥ 150 | Ref | 1.05 | 0.63 | 1.75 | 0.857 | 1.36 | 0.74 | 2.50 | 0.326 | Ref | -1.03 | -18.45 | 16.40 | 0.908 | 14.29 | -7.65 | 36.23 | 0.202 |
|  | HbA1c | % |  |  |  |  |  |  |  |  |  |  | Ref | 0.07 | -0.07 | 0.21 | 0.320 | **0.21** | **0.03** | **0.38** | **0.022** |
|  | Total cholesterol | mg/dL |  |  |  |  |  |  |  |  |  |  | Ref | -1.08 | -7.45 | 5.28 | 0.739 | -4.15 | -12.16 | 3.87 | 0.311 |
|  | LDL cholesterol | mg/dL |  |  |  |  |  |  |  |  |  |  | Ref | 1.98 | -3.48 | 7.45 | 0.477 | -4.17 | -11.05 | 2.71 | 0.235 |
|  | Ankle Brachial Index |  |  |  |  |  |  |  |  |  |  |  | Ref | 0.01 | 0.00 | 0.02 | 0.170 | -0.01 | -0.03 | 0.00 | 0.174 |
|  | Cardio-Ankle Vascular Index |  |  |  |  |  |  |  |  |  |  |  | Ref | -0.04 | -0.19 | 0.11 | 0.618 | **0.17** | **-0.02** | **0.37** | **0.079** |
| Liver | AST | U/L | > 30 | Ref | 0.98 | 0.52 | 1.85 | 0.949 | **2.39** | **1.23** | **4.62** | **0.010** | Ref | 0.13 | -1.31 | 1.57 | 0.862 | **2.54** | **0.73** | **4.36** | **0.006** |
|  | ALT | U/L | > 30 | Ref | 1.31 | 0.77 | 2.25 | 0.321 | **1.89** | **1.02** | **3.52** | **0.044** | Ref | 0.59 | -1.80 | 2.98 | 0.628 | **6.53** | **3.52** | **9.53** | **0.000** |
|  | GGT | U/L | > 50 | Ref | 1.32 | 0.75 | 2.32 | 0.335 | **1.92** | **1.01** | **3.64** | **0.047** | Ref | -1.01 | -7.70 | 5.69 | 0.768 | **8.29** | **-0.14** | **16.72** | **0.054** |
| Inflammation | High Sensitive CRP | mg/dL |  |  |  |  |  |  |  |  |  |  | Ref | 0.00 | -0.04 | 0.05 | 0.848 | 0.03 | -0.03 | 0.09 | 0.364 |
|  | IL-6 | pg/mL |  |  |  |  |  |  |  |  |  |  | Ref | **-0.87** | **-1.77** | **0.04** | **0.060** | -0.74 | -1.88 | 0.40 | 0.204 |
|  | TNF-α | pg/mL |  |  |  |  |  |  |  |  |  |  | Ref | -0.03 | -0.53 | 0.47 | 0.908 | 0.17 | -0.47 | 0.80 | 0.609 |

OR: odds ratio, PD: Periodontal disease, AC: Abdominal circumference, SBP: Systole blood pressure, DBP: Diastole blood pressure, FBG: Fasting blood glucose, HDL-C: HDL cholesterol, TG: Triglycerides , Total-C: Total cholesterol, LDL-C: LDL cholesterol, ABI: Ankle Brachial Index, CAVI: Cardio-Ankle Vascular Index, hs-CRP: High sensitive C-reactive protein, PPD: Probing poket depth.

The OHIP sub-dimensions (SD) evaluate the following evaluate the following:

SD1: Functional limitation, SD2: Physical pain, SD3: Psychological discomfort, SD4: Physical disability, SD5: Psychological disability, SD6: Social disability, SD7: Handicap.

Supplementary Table 7: α-diversity, type (enterotype) and genera in the gut microbiome by orotype

| Category | Variables | Unit or Level | Overall | N type | P type | S type | *p* | *p* corrected |
| --- | --- | --- | --- | --- | --- | --- | --- | --- |
|  | n |  | 644 | 237 | 286 | 121 |  | by bonferroni |
| Alpha diversity | Shannon |  | 1.86±0.18 | 1.86±0.18 | 1.85±0.19 | 1.87±0.17 | 0.893 |  |
|  | Simpson |  | 0.81±0.02 | 0.81±0.02 | 0.81±0.02 | 0.81±0.02 | 0.325 |  |
| Enterotype | Cluster (Two-cluster model) | Cluster 1 | 75.2 (484) | 74.7 (177) | 78.3 (224) | 68.6 (83) | 0.113 |  |
|  |  | Cluster 2 | 24.8 (160) | 25.3 (60) | 21.7 (62) | 31.4 (38) |  |  |
|  | Cluster (Three-cluster model) | Cluster 1 | 58.9 (379) | 57.4 (136) | 59.8 (171) | 59.5 ( 72) | 0.052 |  |
|  |  | Cluster 2 | 24.4 (157) | 24.9 ( 59) | 21.0 ( 60) | 31.4 ( 38) |  |  |
|  |  | Cluster 3 | 16.8 (108) | 17.7 ( 42) | 19.2 ( 55) | 9.1 ( 11) |  |  |
| Genus | Unclassified | % | 8.07±4.66 | 8.36±5 | 7.98±4.44 | 7.72±4.48 | 0.393 | 1 |
|  | *Actinomyces* | % | 0.03±0.04 | 0.02±0.02 | 0.03±0.04 | 0.03±0.05 | 0.001 | **0.032** |
|  | *Bifidobacterium* | % | 6.65±6.61 | 6.55±6.36 | 6.97±7.1 | 6.13±5.89 | 0.440 | 1 |
|  | *Collinsella* | % | 4.75±4.84 | 4.65±5.3 | 4.33±4.45 | 5.85±4.68 | 0.008 | 0.355 |
|  | *Eggerthella* | % | 0.22±0.34 | 0.24±0.35 | 0.23±0.36 | 0.14±0.21 | 0.010 | 0.480 |
|  | *Bacteroides* | % | 12.18±7.85 | 12.08±7.42 | 12.64±8.09 | 11.29±8 | 0.231 | 1 |
|  | *Odoribacter* | % | 0.14±0.32 | 0.12±0.25 | 0.15±0.28 | 0.17±0.49 | 0.436 | 1 |
|  | *Parabacteroides* | % | 1.15±1.55 | 1.14±1.72 | 1.23±1.48 | 1.01±1.35 | 0.366 | 1 |
|  | *Prevotella* | % | 5.85±11.31 | 6.21±11.66 | 5.03±10.51 | 7.05±12.29 | 0.174 | 1 |
|  | *Alistipes* | % | 2.37±3.48 | 2.26±3.4 | 2.46±3.7 | 2.36±3.12 | 0.785 | 1 |
|  | *Gemmiger* | % | 2.18±2.44 | 2.33±2.7 | 2.08±2.42 | 2.13±1.96 | 0.463 | 1 |
|  | *Parasutterella* | % | 0.66±1.31 | 0.72±1.45 | 0.64±1.18 | 0.61±1.32 | 0.668 | 1 |
|  | *Sutterella* | % | 1.2±1.74 | 1.21±1.64 | 1.15±1.59 | 1.26±2.2 | 0.815 | 1 |
|  | *Bilophila* | % | 0.14±0.23 | 0.13±0.25 | 0.14±0.22 | 0.12±0.24 | 0.666 | 1 |
|  | *Escherichia.Shigella* | % | 0.33±1.13 | 0.32±1.13 | 0.35±1.04 | 0.28±1.33 | 0.851 | 1 |
|  | *Haemophilus* | % | 0.09±0.33 | 0.1±0.38 | 0.08±0.29 | 0.07±0.31 | 0.628 | 1 |
|  | *Saccharibacteria_genera_incertae_sedis* | % | 0.02±0.04 | 0.02±0.03 | 0.03±0.04 | 0.01±0.02 | 0.000 | **0.001** |
|  | *Bacillus* | % | 0.04±0.14 | 0.05±0.2 | 0.03±0.08 | 0.03±0.07 | 0.205 | 1 |
|  | *Granulicatella* | % | 0±0.01 | 0±0.01 | 0±0 | 0±0.01 | 0.803 | 1 |
|  | *Lactobacillus* | % | 0.3±1.55 | 0.23±1.82 | 0.28±1.22 | 0.49±1.67 | 0.268 | 1 |
|  | *Streptococcus* | % | 1.46±2.94 | 1.21±2.1 | 1.56±3.44 | 1.67±3.04 | 0.230 | 1 |
|  | *Clostridium.sensu.stricto* | % | 0.14±0.41 | 0.13±0.44 | 0.14±0.32 | 0.15±0.53 | 0.850 | 1 |
|  | *Roseburia* | % | 3.89±3.96 | 3.56±3.58 | 4.23±4.44 | 3.71±3.38 | 0.108 | 1 |
|  | *Dorea* | % | 1.16±1.08 | 1.11±1.04 | 1.14±1.08 | 1.29±1.15 | 0.256 | 1 |
|  | *Anaerostipes* | % | 5.55±5.67 | 5.33±5.33 | 5.57±5.93 | 5.9±5.66 | 0.637 | 1 |
|  | *Coprococcus* | % | 0.45±0.78 | 0.47±0.84 | 0.43±0.79 | 0.45±0.65 | 0.865 | 1 |
|  | *Faecalibacterium* | % | 6.65±5.76 | 6.88±6.55 | 6.7±5.39 | 6.13±4.97 | 0.449 | 1 |
|  | *Ruminococcus* | % | 8.63±6.99 | 8.75±7.33 | 8.63±7.09 | 8.38±6.12 | 0.884 | 1 |
|  | *Phascolarctobacterium* | % | 0.74±1.03 | 0.75±1.1 | 0.71±0.95 | 0.79±1.08 | 0.739 | 1 |
|  | *Dialister* | % | 0.29±0.58 | 0.3±0.57 | 0.3±0.61 | 0.26±0.51 | 0.809 | 1 |
|  | *Veillonella* | % | 0.77±1.74 | 0.75±1.87 | 0.82±1.75 | 0.71±1.44 | 0.786 | 1 |
|  | *Holdemania* | % | 0.01±0.02 | 0.02±0.02 | 0.01±0.02 | 0.02±0.02 | 0.565 | 1 |
|  | *Turicibacter* | % | 0.15±0.48 | 0.2±0.64 | 0.14±0.37 | 0.11±0.3 | 0.164 | 1 |
|  | *Butyricicoccus* | % | 0.76±0.61 | 0.8±0.65 | 0.73±0.57 | 0.79±0.62 | 0.327 | 1 |
|  | *Blautia* | % | 8.55±4.76 | 8.46±4.28 | 8.7±5.26 | 8.38±4.43 | 0.750 | 1 |
|  | *Flavonifractor* | % | 0.23±0.25 | 0.23±0.27 | 0.26±0.26 | 0.18±0.18 | 0.014 | 0.649 |
|  | *Oscillibacter* | % | 0.46±0.64 | 0.48±0.62 | 0.48±0.71 | 0.39±0.51 | 0.354 | 1 |
|  | *Clostridium.XVIII* | % | 0.48±0.86 | 0.51±1.08 | 0.48±0.7 | 0.43±0.72 | 0.649 | 1 |
|  | *Erysipelotrichaceae_incertae_sedis* | % | 0.44±1.15 | 0.5±1.34 | 0.44±1.05 | 0.3±0.99 | 0.257 | 1 |
|  | *Clostridium.XlVa* | % | 0.22±0.28 | 0.24±0.32 | 0.22±0.27 | 0.19±0.21 | 0.265 | 1 |
|  | *Clostridium.XlVb* | % | 0.21±0.38 | 0.21±0.33 | 0.21±0.38 | 0.23±0.47 | 0.859 | 1 |
|  | *Lachnospiracea_incertae_sedis* | % | 2.58±2.13 | 2.69±2.41 | 2.54±1.87 | 2.46±2.14 | 0.545 | 1 |
|  | *Clostridium.IV* | % | 1.77±3.17 | 1.78±3.1 | 1.68±2.78 | 1.95±4.05 | 0.704 | 1 |
|  | *Fusicatenibacter* | % | 2.5±2.52 | 2.56±2.61 | 2.41±2.45 | 2.62±2.49 | 0.635 | 1 |
|  | *Intestinimonas* | % | 0.05±0.11 | 0.05±0.1 | 0.05±0.1 | 0.05±0.13 | 0.985 | 1 |
|  | *Romboutsia* | % | 0.28±0.58 | 0.28±0.52 | 0.28±0.53 | 0.29±0.77 | 0.987 | 1 |
|  | *Intestinibacter* | *%* | 0.13±0.27 | 0.11±0.25 | 0.15±0.32 | 0.09±0.19 | 0.036 | 1 |

Each numerical variable is expressed as mean ± standard deviation, and each categorical variable is expressed as a percentage (N).

p‑value was calculated by the Kruskal-Wallis test (numerical variables) and Fisher’s exact test (categorical variables). Red numbers represent p<0.05.

Supplementary Table 8: Coeffficients corresponding to each bacterial genus in the orotype classification model

| Community | Genus | P type | S type |
| --- | --- | --- | --- |
|  | (Intercept) | -27.53 | -71.50 |
| 1 | *Neisseria* | -289.52 | -190.06 |
|  | *Fusobacterium* | -203.67 | -115.99 |
|  | *Porphyromonas* | -154.10 | -237.65 |
|  | *SR1_genera_incertae_sedis* | -87.97 | -267.05 |
|  | *Capnocytophaga* | -49.64 | -16.88 |
|  | *Parvimonas* | -38.21 | -2.29 |
|  | Unclassified | -17.83 | -285.54 |
|  | *Peptostreptococcus* | -4.75 | -18.22 |
|  | *Catonella* | -4.17 | -1.63 |
|  | *Peptococcus* | 5.01 | -1.42 |
|  | *Veillonella* | 229.74 | 300.83 |
| 2 | *Granulicatella* | -212.12 | 208.05 |
|  | *Gemella* | -72.19 | 75.29 |
|  | *Haemophilus* | -9.34 | 63.88 |
|  | *Mogibacterium* | 0.97 | -1.17 |
|  | *Centipeda* | 3.76 | -1.36 |
|  | *Atopobium* | 71.12 | 98.14 |
|  | *Campylobacter* | 75.31 | -44.29 |
|  | *Megasphaera* | 97.45 | -4.71 |
|  | *Prevotella* | 148.68 | 7.39 |
|  | *Actinomyces* | 165.25 | 276.89 |
| 3 | *Saccharibacteria_genera_incertae_sedis* | -25.38 | -140.72 |
|  | *Eubacterium* | -1.97 | -19.59 |
|  | *Solobacterium* | 27.04 | -22.75 |
|  | *Rothia* | 27.62 | 306.82 |
|  | *Streptococcus* | 111.42 | 183.75 |
| 4 | *Selenomonas* | -3.66 | 0.68 |
|  | *Anaeroglobus* | -3.28 | 0.57 |
|  | *Olsenella* | 0.75 | -5.96 |
|  | *Treponema* | 0.82 | -21.62 |
|  | *Tannerella* | 5.48 | -7.20 |
|  | *Dialister* | 5.86 | -1.53 |
|  | *Slackia* | 6.20 | -2.04 |
| 5 | *Butyrivibrio* | 3.76 | -2.65 |
|  | *Stomatobaculum* | 4.33 | -34.07 |
|  | *Leptotrichia* | 28.78 | -16.87 |
|  | *Lachnoanaerobaculum* | 99.63 | -61.27 |
| Others | *Corynebacterium* | -41.59 | 16.21 |
|  | *Aggregatibacter* | -30.20 | 0.79 |
|  | *Schlegelella* | -6.35 | 5.52 |
|  | *Cardiobacterium* | -5.96 | 0.93 |
|  | *Lautropia* | -5.74 | 0.62 |
|  | *Morococcus* | -1.58 | 1.13 |
|  | *Abiotrophia* | -0.87 | 6.79 |
|  | *Kingella* | 1.00 | -0.29 |
|  | Other Genera | 16.89 | -31.24 |
|  | *Alloprevotella* | 40.40 | -58.67 |
|  | *Oribacterium* | 65.30 | -11.06 |

Supplementary Table 9: Associations between the transition of orotypes and the change of lifestyle factors and health status

|  | 2016 |  | N type | | | |  | P type | | | |  | S type | | | |  |
| --- | --- | --- | --- | --- | --- | --- | --- | --- | --- | --- | --- | --- | --- | --- | --- | --- | --- |
|  | 2019 | Unit/Level | Overall | N type | P type | S type | *p* | Overall | N type | P type | S type | *p* | Overall | N type | P type | S type | *p* |
| n |  |  | 102 | 61 | 32 | 9 |  | 211 | 38 | 152 | 21 |  | 64 | 8 | 21 | 35 |  |
| Sex |  | Male | 41.2 (42) | 45.9 (28) | 37.5 (12) | 22.2 (2) | 0.354 | 39.8 ( 84) | 44.7 (17) | 38.2 ( 58) | 42.9 ( 9) | 0.726 | 46.9 (30) | 50.0 (4) | 33.3 ( 7) | 54.3 (19) | 0.309 |
|  |  | Female | 58.8 (60) | 54.1 (33) | 62.5 (20) | 77.8 (7) |  | 60.2 (127) | 55.3 (21) | 61.8 ( 94) | 57.1 (12) |  | 53.1 (34) | 50.0 (4) | 66.7 (14) | 45.7 (16) |  |
| Age |  | Years | 52.4±12.2 | 51.4±12.6 | 52.8±12.3 | 57.1±8 | 0.411 | 50.1±13.4 | 49.6±12.6 | 50.9±13.2 | 45±15.6 | 0.158 | 51.8±13.9 | 48.8±9.9 | 51.5±13.3 | 52.7±15.2 | 0.771 |
| Smoking | 2016 | Never | 69.6 (71) | 70.5 (43) | 65.6 (21) | 77.8 (7) | 0.823 | 55.9 (118) | 60.5 (23) | 54.6 ( 83) | 57.1 (12) | 0.56 | 50.0 (32) | 75.0 (6) | 33.3 ( 7) | 54.3 (19) | 0.243 |
|  |  | Current | 5.9 ( 6) | 4.9 ( 3) | 6.2 ( 2) | 11.1 (1) |  | 19.0 ( 40) | 13.2 ( 5) | 19.1 ( 29) | 28.6 ( 6) |  | 31.2 (20) | 25.0 (2) | 42.9 ( 9) | 25.7 ( 9) |  |
|  |  | Past | 24.5 (25) | 24.6 (15) | 28.1 ( 9) | 11.1 (1) |  | 25.1 ( 53) | 26.3 (10) | 26.3 ( 40) | 14.3 ( 3) |  | 18.8 (12) | 0.0 (0) | 23.8 ( 5) | 20.0 ( 7) |  |
|  | 2019 | Never | 72.5 (74) | 68.9 (42) | 75.0 (24) | 88.9 (8) | 0.453 | 58.8 (124) | 65.8 (25) | 57.9 ( 88) | 52.4 (11) | 0.523 | 56.2 (36) | 62.5 (5) | 52.4 (11) | 57.1 (20) | 0.794 |
|  |  | Current | 5.9 ( 6) | 4.9 ( 3) | 6.2 ( 2) | 11.1 (1) |  | 19.9 ( 42) | 10.5 ( 4) | 21.1 ( 32) | 28.6 ( 6) |  | 25.0 (16) | 12.5 (1) | 33.3 ( 7) | 22.9 ( 8) |  |
|  |  | Past | 21.6 (22) | 26.2 (16) | 18.8 ( 6) | 0.0 (0) |  | 21.3 ( 45) | 23.7 ( 9) | 21.1 ( 32) | 19.0 ( 4) |  | 18.8 (12) | 25.0 (2) | 14.3 ( 3) | 20.0 ( 7) |  |
| Sugar and sweeteners | 2016 | g/day | 4.7±4 | 5.3±4 | 4.1±3.7 | 2.8±3.8 |  | 6±4.9 | 6.8±5 | 5.7±4.9 | 6.8±5.1 | 0.342 | 4.9±4.6 | 4±5.4 | 4.2±3.8 | 5.6±4.9 | 0.457 |
|  | 2019 |  | 4.3±3.8 | 4.1±3.5 | 5±3.9 | 3.6±5.2 |  | 5.1±4.3 | 4.9±3.7 | 5±4.5 | 6±4 | 0.574 | 4.3±3.3 | 2.4±2.2 | 3.9±1.9 | 4.9±4 | 0.123 |
|  | Δ2019-2016 |  | -0.4±3.9 | **-1.2±3.9** | **0.8±3.1** | **0.8±5.2** | **0.031** | -1±4.2 | -2±3.9 | -0.7±4.3 | -0.8±4.8 | 0.278 | -0.6±4.6 | -1.5±3.6 | -0.2±3.6 | -0.6±5.4 | 0.807 |
| Vegetables | 2016 | g/day | 197.3±118.3 | 190.7±118.5 | 194.7±109 | 251.6±147.3 |  | 169.8±100.8 | 188.3±124.2 | 169±98.5 | 141.8±57.8 |  | 173.7±109.6 | 139.6±115.9 | 173.6±94.4 | 181.5±118 |  |
|  | 2019 |  | 202.8±126.5 | 209.8±141.6 | 202.4±103.4 | 156.9±85.9 |  | 172.4±106.8 | 192.3±96 | 169.8±108.6 | 155.5±112.1 |  | 196.8±118.6 | 149.2±90.2 | 208.6±124.4 | 200.6±121.1 |  |
|  | Δ2019-2016 |  | 5.5±126.8 | **19±135.4** | **7.8±104.2** | **-94.6±104.4** | **0.041** | 2.6±110.4 | 4±113 | 0.7±107.5 | 13.7±130.6 | 0.878 | 23.1±112.9 | 9.6±77.4 | 35±82.4 | 19.1±135 | 0.826 |
| Fruits | 2016 | g/day | 98.8±96.9 | 80.9±81.4 | 125.1±123.1 | 126.5±63.8 |  | 79.9±80.2 | 66.3±80 | 84±83 | 74.4±56.1 |  | 82.7±79.1 | 34±36.5 | 86.9±82.2 | 91.2±81.9 |  |
|  | 2019 |  | 90.4±75 | 85.8±77.8 | 101.1±76.4 | 82.8±49.1 |  | 81±80.1 | 80±74.2 | 80±80.7 | 90.2±88.3 |  | 76.1±65.3 | 59.4±47.5 | 72.1±60.6 | 82.4±71.8 |  |
|  | Δ2019-2016 |  | -8.5±80.6 | 4.9±75.6 | -24.1±92.9 | -43.7±46.4 | **0.1** | 1.1±84.3 | 13.6±61.8 | -4±91.3 | 15.8±62.3 | 0.364 | -6.5±72.8 | 25.4±57.7 | -14.8±68.1 | -8.9±78.2 | 0.403 |
| Mushrooms | 2016 | g/day | 10.6±9.1 | 10.4±9.2 | 11±9.3 | 10.4±9.4 |  | 10±9.2 | 10.2±6.9 | 10.2±10.1 | 8.5±6.1 |  | 9.1±7.7 | 8.8±8.3 | 7.3±5.2 | 10.3±8.7 |  |
|  | 2019 |  | 11.8±11.7 | 11.5±10.6 | 12.7±11 | 10.8±20.4 |  | 9.9±9.2 | 10.3±7.5 | 10.2±9.8 | 7.2±7.6 |  | 11.3±10.4 | 11.2±8.1 | 10.7±7.7 | 11.7±12.3 |  |
|  | Δ2019-2016 |  | 1.2±11 | 1.1±8.6 | 1.7±13 | 0.4±17.9 | 0.944 | -0.1±8.6 | 0.1±8 | 0±9 | -1.3±7.1 | 0.799 | 2.2±9.8 | 2.4±6 | 3.4±7.1 | 1.4±11.8 | 0.762 |
| Snacks | 2016 | g/day | 45.5±40.2 | 43±44.4 | 50±34.3 | 45.6±30.4 |  | 45.9±33.7 | 39.2±29.3 | 48.6±35.5 | 38.7±24.4 |  | 47.2±41.6 | 41.4±26.2 | 37.8±26.6 | 54.3±50.3 |  |
|  | 2019 |  | 40.7±29.9 | 39.4±31.8 | 41.6±28.5 | 46.3±22 |  | 45.3±35 | 37.1±29.8 | 49.1±36.8 | 32.7±23.2 |  | 42.5±29.6 | 38.6±35.2 | 40.9±23.9 | 44.3±32 |  |
|  | Δ2019-2016 |  | -4.7±30.1 | -3.6±27.9 | -8.4±35 | 0.7±27.9 | 0.659 | -0.6±32.2 | -2.1±28.7 | 0.6±34.7 | -6±14.2 | 0.649 | -4.8±32.4 | -2.9±20.7 | 3.1±25.2 | -10±37.7 | 0.344 |
| Discretionary beverages | 2016 | g/day | 641.5±347.9 | 693±368.9 | 553.3±324.6 | 605.8±216.5 |  | 693.8±443.9 | 654.7±402.8 | 696.2±412.5 | 746.9±691.6 |  | 798.9±441.5 | 755±375.5 | 962.8±511.6 | 710.6±391.2 |  |
|  | 2019 |  | 688.7±376.6 | 705.6±370.6 | 644.9±398.7 | 729.5±364.7 |  | 650.2±360.9 | 571.1±308.5 | 668±343.3 | 664.9±535.7 |  | 733.7±428.1 | 816.2±366 | 714.9±488.3 | 726.1±412.2 |  |
|  | Δ2019-2016 |  | 47.2±297.6 | 12.6±291.7 | 91.6±308.7 | 123.7±295.4 | 0.348 | -43.6±320.5 | -83.7±330 | -28.2±320 | -82±312.8 | 0.538 | **-65.2±349.8** | **61.3±194.6** | **-247.9±330.1** | **15.5±351.7** | **0.011** |
| Brushing | 2016 | g/day | 2.2±0.8 | 2.2±0.9 | 2.3±0.6 | 2.2±1 |  | 2.2±0.8 | 2.4±0.7 | 2.1±0.7 | 2.1±1.1 |  | 2.1±0.7 | 2±0.5 | 2.2±0.8 | 2.1±0.8 |  |
|  | 2019 |  | 2.3±0.8 | 2.2±0.9 | 2.3±0.7 | 2.4±1 |  | 2.3±0.8 | 2.5±0.7 | 2.2±0.8 | 2.4±0.9 |  | 2.2±0.7 | 2±0.5 | 2.4±0.8 | 2.1±0.7 |  |
|  | Δ2019-2016 |  | 0±0.6 | 0±0.6 | 0.1±0.4 | 0.2±1 | 0.491 | 0.1±0.6 | 0.1±0.5 | 0.1±0.6 | 0.3±0.7 | 0.298 | 0±0.5 | 0±0 | 0.2±0.5 | -0.1±0.5 | 0.16 |
| Teeth number | 2016 | /day | 25.6±4.5 | 26.1±4 | 25.2±3.9 | 23.4±8.6 |  | 24.3±6.9 | 25.1±4.7 | 24.3±6.9 | 22.5±10.1 |  | 23.9±7 | 26.6±1.7 | 23.7±6 | 23.4±8.2 |  |
|  | 2019 |  | 25.1±5.4 | 26.1±4.3 | 23.9±6 | 23±8.4 |  | 23.8±7.3 | 24.5±5.5 | 23.9±7.1 | 21.9±10.7 |  | 23.6±7.3 | 27±1.2 | 23.7±6.1 | 22.7±8.5 |  |
|  | Δ2019-2016 |  | -0.4±2.8 | 0±1.2 | -1.3±4.6 | -0.4±0.7 | 0.115 | -0.4±2.1 | -0.6±2.2 | -0.4±2.1 | -0.6±1.5 | 0.777 | -0.3±1.7 | 0.4±0.5 | 0±1.7 | -0.7±1.8 | 0.133 |
| Caries | 2016 | number | 0.5±1 | 0.4±0.8 | 0.6±1.2 | 0.8±1.4 |  | 0.7±1.5 | 0.7±1.1 | 0.7±1.7 | 0.3±0.6 |  | 0.5±1.1 | 0.1±0.4 | 0.7±1.5 | 0.5±0.9 |  |
|  | 2019 |  | 0.4±0.9 | 0.3±0.7 | 0.7±1 | 0.3±1 |  | 0.6±1.4 | 0.3±0.6 | 0.6±1.6 | 0.5±0.8 |  | 0.4±1 | 0±0 | 0.8±1.3 | 0.3±0.8 |  |
|  | Δ2019-2016 |  | -0.1±0.9 | -0.1±0.8 | 0.1±1 | -0.4±1.4 | 0.276 | -0.1±1.1 | -0.4±1.2 | 0±1.1 | 0.2±1 | 0.112 | 0±1.2 | -0.1±0.4 | 0.1±1.9 | -0.1±0.8 | 0.694 |
| Periodontal disease | 2016 | Yes % | 49.0 (50) | 49.2 (30) | 53.1 (17) | 33.3 (3) | 0.781 | 48.8 (103) | 42.1 (16) | 52.0 (79) | 38.1 (8) | 0.371 | 48.4 (31) | 37.5 (3) | 42.9 (9) | 54.3 (19) | 0.279 |
|  | 2019 |  | 29.4 (30) | 21.3 (13) | 46.9 (15) | 22.2 (2) | **0.091** | 38.0 (81) | 36.8 (14) | 40.8 (62) | 19.0 (4) | 0.343 | 26.5 (17) | 25.0 (2) | 19.0 (4) | 31.4 (11) | 0.354 |
| OHIP14 | 2016 |  | 4.5±6.1 | 3.3±5.5 | 5.7±6.8 | 8.4±5.5 |  | 4.4±6.6 | 5.4±6.7 | 4.1±6.4 | 5.2±7.8 |  | 5.1±7.4 | 5.6±8.4 | 4.9±7.8 | 5.1±7.2 |  |
|  | 2019 |  | 3.6±5.9 | 2.8±5.6 | 4.8±6.8 | 4.7±4 |  | 3.7±6.1 | 2.5±5.1 | 3.7±5.9 | 5.5±8.1 |  | 5.5±6.7 | 3±4.7 | 5.9±7.7 | 5.9±6.6 |  |
|  | Δ2019-2016 |  | -0.9±6.2 | -0.4±5.9 | -0.8±6.7 | -3.8±5.6 | 0.32 | -0.8±6.8 | -2.9±7.1 | -0.4±6.7 | 0.3±5.9 | 0.091 | 0.4±7.2 | -2.6±6.6 | 1±8 | 0.8±6.9 | 0.445 |
| AC | 2016 | cm | 82.5±9 | 81.8±8.6 | 83.7±9.6 | 82.6±9.6 |  | 83.7±8.7 | 83±8.4 | 84±9 | 83±6.8 |  | 85.3±9.2 | 78.6±9.8 | 84.1±9 | 87.5±8.6 |  |
|  | 2019 |  | 83.2±9.4 | 82.3±9.5 | 85±8.9 | 83±10.1 |  | 84.3±8.9 | 84±9.5 | 84.6±9.1 | 82.3±6.7 |  | 85.6±9.5 | 79.8±10.3 | 84.1±9.8 | 87.9±8.5 |  |
|  | Δ2019-2016 |  | 0.7±3.1 | 0.5±3.2 | 1.3±2.9 | 0.4±3 | 0.493 | 0.5±3.7 | 1±3.8 | 0.6±3.7 | -0.7±3.4 | 0.248 | 0.4±4.4 | 1.2±4.5 | 0±4.7 | 0.4±4.4 | 0.795 |
| HbA1c | 2016 | % | 5.8±0.4 | 5.8±0.4 | 5.7±0.5 | 6±0.6 |  | 5.8±0.6 | 5.9±0.6 | 5.8±0.6 | 5.6±0.5 |  | 5.9±0.9 | 5.7±0.2 | 6±1 | 6±1 |  |
|  | 2019 |  | 5.7±0.6 | 5.6±0.4 | 5.7±0.8 | 6±0.8 |  | 5.7±0.6 | 5.7±0.5 | 5.7±0.7 | 5.6±0.4 |  | 5.8±0.9 | 5.5±0.5 | 6±1.1 | 5.8±0.8 |  |
|  | Δ2019-2016 |  | -0.1±0.3 | -0.1±0.2 | 0±0.3 | 0±0.3 | 0.122 | -0.1±0.3 | -0.2±0.4 | -0.1±0.3 | 0±0.2 | **0.095** | -0.1±0.4 | -0.2±0.5 | 0±0.3 | -0.1±0.3 | 0.331 |
| HDL | 2016 | mg/dL | 68.1±15 | 69.9±14.5 | 64.3±16 | 69.3±14.3 |  | 64.2±17.4 | 65±12.7 | 64.1±19 | 63.9±12.1 |  | 64.5±18.1 | 75.6±20.9 | 65±17 | 61.8±17.6 |  |
|  | 2019 |  | 67.6±16.2 | 70.3±15.8 | 62.2±17.3 | 68.9±11.2 |  | 64.7±16.8 | 65.2±14.8 | 64.4±17.7 | 65.8±13.7 |  | 63.8±18.1 | 73.4±19.2 | 66±16.2 | 60.3±18.4 |  |
|  | Δ2019-2016 |  | -0.5±8 | 0.4±8.9 | -2.2±6.6 | -0.4±6.4 | 0.346 | 0.5±9.4 | 0.2±10 | 0.3±9.3 | 2±9.1 | 0.743 | -0.7±9.1 | -2.3±10 | 1.1±10.2 | -1.4±8.3 | 0.537 |
| AST | 2016 | U/L | 22.4±7.7 | 22.7±7.8 | 22.2±8.1 | 21±5.6 |  | 22.9±9.4 | 23.9±9.5 | 22.9±9.6 | 21.1±7.7 |  | 23.1±7.3 | 26.3±7.2 | 23.7±7.9 | 22±6.9 |  |
|  | 2019 |  | 21.5±6 | 22±6.1 | 20.4±5.1 | 22.2±8.6 |  | 21.6±6.6 | 21.8±7.8 | 21.6±6.5 | 21.3±5.5 |  | 21.9±7.6 | 26.5±13.3 | 21.8±5.7 | 20.8±6.8 |  |
|  | Δ2019-2016 |  | -0.9±5.7 | -0.8±5.2 | -1.8±6.9 | 1.2±4.5 | 0.357 | -1.4±8.1 | -2.2±5.3 | -1.4±9 | 0.2±5.4 | 0.57 | -1.3±7.9 | 0.3±10.6 | -1.9±6.5 | -1.2±8.2 | 0.812 |
| ALT | 2016 | U/L | 20.6±13.7 | 20.9±14 | 21.2±14.3 | 17.2±8.2 |  | 22.7±14 | 23.3±11.3 | 23.1±15.1 | 19±9.2 |  | 21.6±11.4 | 21.6±6.7 | 23.7±13.7 | 20.3±10.8 |  |
|  | 2019 |  | 19.4±10.2 | 19.3±9.6 | 20.1±11.9 | 17.6±7.8 |  | 20.9±12.3 | 21.1±10.3 | 21±13.1 | 20.1±10.6 |  | 20.1±10.8 | 24.8±17.2 | 22±11.3 | 17.9±8.3 |  |
|  | Δ2019-2016 |  | -1.2±9.3 | -1.5±8.7 | -1±11.2 | 0.3±5.8 | 0.848 | -1.8±12.6 | -2.3±8.7 | -2.1±13.8 | 1.1±8.2 | 0.525 | -1.5±10.9 | 3.1±14.9 | -1.7±9 | -2.4±10.9 | 0.435 |
| GGT | 2016 | U/L | 32.6±31.6 | 37±38 | 27.8±18.3 | 20.1±8.3 |  | 30.4±28.6 | 34.7±37.6 | 29.2±25 | 31.2±34.9 |  | 33.3±25.5 | 41.1±46.4 | 33.6±23.7 | 31.4±20.3 |  |
|  | 2019 |  | 33.6±32.6 | 37±38.2 | 30.2±22.7 | 23±13.4 |  | 28.2±22.6 | 29.2±22.1 | 28.3±23.4 | 25.9±17.5 |  | 33.6±32.7 | 49.4±66.2 | 31.1±26.3 | 31.5±24.7 |  |
|  | Δ2019-2016 |  | 1±17.9 | 0±20.2 | 2.5±14.3 | 2.9±13.5 | 0.781 | -2.1±14.8 | -5.6±21 | -0.8±11.5 | -5.3±20.9 | 0.121 | 0.3±18.3 | 8.3±20.8 | -2.5±10.1 | 0.1±21.3 | 0.374 |

Supplementary Table 10: Nutrient intakes by orotype

|  | Unit | Overall | N type | P type | S type | *p* |
| --- | --- | --- | --- | --- | --- | --- |
| n |  | 644 | 237 | 286 | 121 |  |
| Protein | g/day | 73.1±16 | 76.1±17 | 71.5±14.8 | 70.8±16.2 | 0.001 |
| Lipid | g/day | 68±20.2 | 71.3±21.5 | 66.5±18 | 65±21.9 | 0.005 |
| Saturated fatty acids | g/day | 21.3±8.7 | 22.3±9.3 | 20.9±8.1 | 20.3±8.8 | 0.07 |
| Monounsaturated fatty acids | g/day | 25.4±8.8 | 26.7±10.1 | 24.7±7.4 | 24.2±8.7 | 0.009 |
| Polyunsaturated fatty acids | g/day | 14±4.6 | 14.5±4.6 | 13.8±4.6 | 13.4±4.6 | 0.047 |
| n-3 Polyunsaturated fatty acids (Omega-3) | g/day | 2.2±0.9 | 2.3±0.9 | 2.2±0.8 | 2.2±0.9 | 0.224 |
| n-6 Polyunsaturated fatty acids (Omega-6) | g/day | 11.7±3.9 | 12.2±3.9 | 11.5±3.9 | 11.1±3.9 | 0.04 |
| Cholesterol | mg/day | 332.9±255 | 370±303.7 | 300.6±175.5 | 336.4±297.9 | 0.008 |
| Carbohydrate | g/day | 269.9±53.6 | 259.4±57.1 | 277.2±49.1 | 273.2±53.9 | 0.001 |
| Monosaccharide equivalent | g/day | 228±54.9 | 220.8±56.2 | 233.9±51.6 | 228.3±58.6 | 0.025 |
| Total dietary fiber | g/day | 13.4±5.6 | 13.9±6 | 13.5±5.6 | 12.1±5 | 0.018 |
| Soluble dietary fiber | g/day | 3.3±1.6 | 3.4±1.6 | 3.3±1.6 | 2.9±1.3 | 0.036 |
| Insoluble dietary fiber | g/day | 9.6±4 | 9.9±4.2 | 9.7±3.9 | 8.7±3.6 | 0.024 |
| Ash | g/day | 18.4±5 | 19.2±5.1 | 18.3±5 | 17.2±4.7 | 0.001 |
| Sodium | mg/day | 3787.8±1227.6 | 3941.7±1293.7 | 3771.9±1198.1 | 3524.1±1121 | 0.009 |
| Potassium | mg/day | 2845.7±932.2 | 2944.8±924.9 | 2847.4±971.6 | 2647.6±820.6 | 0.017 |
| Calcium | mg/day | 636.4±451.8 | 681.9±484.6 | 623.2±451.6 | 578.5±373.7 | 0.099 |
| Magnesium | mg/day | 324.5±105.2 | 332±98.7 | 325.3±118.1 | 307.7±81.1 | 0.115 |
| Phosphorus | mg/day | 1160±359.6 | 1211.5±396 | 1139.2±333 | 1108.2±334.9 | 0.015 |
| Iron | mg/day | 8.3±2.5 | 8.7±2.6 | 8.1±2.4 | 8±2.5 | 0.007 |
| Zinc | mg/day | 8.7±1.7 | 9±1.8 | 8.5±1.7 | 8.3±1.8 | 0.001 |
| Copper | mg/day | 1.2±0.4 | 1.3±0.4 | 1.2±0.4 | 1.2±0.3 | 0.17 |
| Manganese | mg/day | 3.2±1.2 | 3.2±1.4 | 3.2±1.1 | 3.2±1.2 | 0.805 |
| Iodine | μg/day | 201.2±220.2 | 225±248.8 | 196±211.4 | 166.8±171.6 | 0.053 |
| Selenium | μg/day | 82.8±26.4 | 86.9±29.1 | 79.2±22.6 | 83.5±28.1 | 0.004 |
| Chromium | μg/day | 7.5±4 | 7.3±3.3 | 7.9±4.4 | 7±4.1 | 0.085 |
| Molybdenum | μg/day | 225.6±133.1 | 230.3±127.7 | 226.5±152.9 | 214.3±84.3 | 0.553 |
| α-Carotene | μg/day | 473.5±518.9 | 526.8±584.4 | 456.4±491.8 | 409.3±431.8 | 0.097 |
| β-Carotene | μg/day | 2324.6±1843.3 | 2548.5±1889.8 | 2275.8±1922.5 | 2001.3±1481.1 | 0.024 |
| β-Cryptoxanthin | μg/day | 495.4±934.2 | 398.2±735.5 | 622.8±1151.3 | 384.8±628 | 0.008 |
| Lycopene | μg/day | 4591.4±7074.3 | 5500.2±8051.8 | 3843.6±6593.3 | 4579±5869.9 | 0.028 |
| Retinol | μg/day | 323.9±366.8 | 347.4±386.9 | 302.3±348.8 | 328.8±367.7 | 0.371 |
| Vitamin D | μg/day | 7±4.7 | 7.2±5 | 6.7±4.1 | 7.4±5.4 | 0.249 |
| α-Tocopherol | mg/day | 7.8±3.1 | 8.1±3.4 | 7.8±2.9 | 7.4±2.9 | 0.062 |
| β-Tocopherol | mg/day | 0.4±0.2 | 0.5±0.2 | 0.4±0.2 | 0.4±0.2 | 0.326 |
| γ-Tocopherol | mg/day | 12±6 | 12.3±6.3 | 12±6 | 11.2±5.5 | 0.269 |
| δ-Tocopherol | mg/day | 2.9±1.9 | 3±1.9 | 2.8±2.1 | 2.6±1.5 | 0.212 |
| Vitamin K | μg/day | 291.6±269.5 | 314.8±270.9 | 285.2±296.9 | 261.4±182.9 | 0.179 |
| Vitamin B1 (Thiamine) | mg/day | 0.9±0.3 | 1±0.3 | 0.9±0.2 | 0.9±0.3 | 0.004 |
| Vitamin B2 (Riboflavin) | mg/day | 1.5±0.7 | 1.6±0.7 | 1.4±0.7 | 1.4±0.6 | 0.034 |
| Niacin (Vitamin B3) | mg/day | 20±6.7 | 19.6±5.9 | 20.4±7.5 | 19.7±6.4 | 0.34 |
| Vitamin B6 | mg/day | 1.3±0.3 | 1.4±0.3 | 1.3±0.4 | 1.3±0.4 | 0.04 |
| Vitamin B12 | μg/day | 6.5±4.8 | 6.9±6.3 | 6.1±3.5 | 6.5±4 | 0.146 |
| Folate (Vitamin B9) | μg/day | 339.1±137.3 | 358.6±146.5 | 327.9±135 | 327.3±119.9 | 0.022 |
| Pantothenic acid (Vitamin B5) | mg/day | 7.2±2.4 | 7.5±2.6 | 7±2.5 | 6.9±2 | 0.026 |
| Biotin (Vitamin B7) | μg/day | 50.3±20.3 | 52.7±22 | 48.7±18 | 49.7±21.8 | 0.074 |
| Vitamin C | mg/day | 92.6±61.6 | 96.2±57.4 | 93.1±68 | 84.4±52.6 | 0.225 |
| Salt equivalent | g/day | 9.6±3.1 | 10±3.3 | 9.5±3 | 8.9±2.9 | 0.009 |
| Alcohol | g/day | 16.6±29.4 | 16.6±29.2 | 15.5±27.7 | 19.4±33.6 | 0.476 |
| Nitrate ion | g/day | 0.1±0.1 | 0.1±0.1 | 0.1±0.1 | 0.1±0.1 | 0.043 |
| Theobromine | g/day | 0±0 | 0±0 | 0±0 | 0±0 | 0.022 |
| Caffeine | g/day | 0.3±0.4 | 0.3±0.3 | 0.4±0.4 | 0.3±0.3 | 0.033 |
| Tannin | g/day | 1±1.1 | 0.9±1 | 1.1±1.3 | 0.9±1 | 0.043 |
| Polyphenol | g/day | 0±0.1 | 0±0.1 | 0.1±0.1 | 0±0.1 | 0.022 |
| Acetic acid | g/day | 0.1±0.1 | 0.1±0.1 | 0.1±0.1 | 0.1±0.1 | 0.753 |
| Organic acids | g/day | 1.4±1 | 1.4±1 | 1.4±1 | 1.3±0.8 | 0.521 |
| Daidzein | mg/day | 18.3±21 | 20.2±20.3 | 17.5±23.9 | 16.3±13.7 | 0.181 |
| Genistein | mg/day | 30.5±35.7 | 33.8±35.6 | 29.2±39.8 | 27±23.8 | 0.168 |

Each variable is expressed as mean ± standard deviation. p‑value was calculated by the Kruskal-Wallis test.

Supplementary Table 11: List of measurement items and its methods

| Category | Variables | Method | Detail |
| --- | --- | --- | --- |
| Subject attributes | Age | Questionnnaire | Numeric |
|  | Sex | Questionnnaire | Female, Male |
|  | Educational background | Questionnnaire | Primary School, Junior High School, High School, Technical School, College |
|  | Cohabits number | Questionnnaire | Numeric |
|  | Spouse | Questionnnaire | Yes, No, Bereavement, Divorce |
| Lifestyle | Smoking | Questionnnaire | Never, Past, Current |
|  | Passive smoking | Questionnnaire | Yes, No |
|  | Drinking | Questionnnaire | Never, Past, Current |
|  | Alcoholic beverage | FFQ | Adustment using residual method |
|  | Exercise | Questionnnaire | Everyday, 5-6/week, 2-4/ week,1 or less/week, No |
|  | Sports | Questionnnaire | Yes, No |
|  | Sleep | Japanese version of the Epworth Sleepiness Scal |  |
| Dietary habits | Grains | FFQ | Adustment using residual method |
|  | Potato | FFQ | Adustment using residual method |
|  | Sugar and sweeteners | FFQ | Adustment using residual method |
|  | Pulse | FFQ | Adustment using residual method |
|  | Nuts and bolts | FFQ | Adustment using residual method |
|  | Vegetables | FFQ | Adustment using residual method |
|  | Green and yellow vegetables | FFQ | Adustment using residual method |
|  | Other vegetables | FFQ | Adustment using residual method |
|  | Fruits | FFQ | Adustment using residual method |
|  | Mushrooms | FFQ | Adustment using residual method |
|  | Seaweed | FFQ | Adustment using residual method |
|  | Seafood | FFQ | Adustment using residual method |
|  | Meats | FFQ | Adustment using residual method |
|  | Eggs | FFQ | Adustment using residual method |
|  | Dairy | FFQ | Adustment using residual method |
|  | Oils and vats | FFQ | Adustment using residual method |
|  | Snacks | FFQ | Adustment using residual method |
|  | Discretionary beverages | FFQ | Adustment using residual method |
|  | Seasoning and spices | FFQ | Adustment using residual method |
| Oral care | Brushing | Questionnnaire | Numeric |
|  | Dental clinic for treatment | Questionnnaire | No, Yes (Last one year) |
|  | Dental clinic for checkup or cleaning | Questionnnaire | No, Yes (Last one year) |
| Medical history | Hypertension | Questionnnaire | Never, Yes and currently taking medication (Current), Yes currently taking no medication (Past) |
|  | Diabetes | Questionnnaire | Never, Yes and currently taking medication (Current), Yes currently taking no medication (Past) |
|  | Hyperlipidemia | Questionnnaire | Never, Yes and currently taking medication (Current), Yes currently taking no medication (Past) |
|  | Osteoporosis | Questionnnaire | Never, Yes and currently taking medication (Current), Yes currently taking no medication (Past) |
|  | Bronchial asthma | Questionnnaire | Never, Yes and currently taking medication (Current), Yes currently taking no medication (Past) |
|  | Depression | Questionnnaire | Never, Yes and currently taking medication (Current), Yes currently taking no medication (Past) |
|  | Dementia | Questionnnaire | Never, Yes and currently taking medication (Current), Yes currently taking no medication (Past) |
| Oral health | Teeth Number | Clinical checkup | Numeric |
|  | Eichner | Clinical checkup | A1–C3 |
|  | Caries | Clinical checkup | Numeric |
|  | Periodontal disease | Clinical checkup | Perodontal Poket Depth < 4mm, 4-6mm, >6mm |
|  | OHIP14 | Oral Health Impact Profile |  |
|  | Salivary IgA | Saliva |  |
|  | Salivation | Saliva |  |
| MetS | Abdominal circumference | Measuring tape |  |
|  | Systole blood pressure | Sphygmomanometer | HEM-1040(OMRON Corp., Kyoto, Japan) or ES-P2000BR (Terumo Corp., Tokyo, Japan) |
|  | Diastole blood pressure | Sphygmomanometer | HEM-1040(OMRON Corp., Kyoto, Japan) or ES-P2001BR (Terumo Corp., Tokyo, Japan) |
|  | Fasting blood glucose | Glucokinase-glucose-6-phosphate dehydrogenase method | Measurements were performed by LSI Medience Corp. |
|  | HDL cholesterol | Enzymatic method | Measurements were performed by LSI Medience Corp. |
|  | Triglycerides | Enzymatic method | Measurements were performed by LSI Medience Corp. |
|  | HbA1c | Enzymatic method | Measurements were performed by LSI Medience Corp. (Tokyo, Japan, a clinical laboratory company) |
|  | Total cholesterol | Enzymatic method | Measurements were performed by LSI Medience Corp. |
|  | LDL cholesterol | Enzymatic method | Measurements were performed by LSI Medience Corp. |
|  | Ankle Brachial Index (ABI) | Network arterial stiffness detection device | BP-203RPEⅢ (OMRON Corp.) |
|  | Cardio-Ankle Vascular Index (CAVI) | Network arterial stiffness detection device | BP-204RPEⅢ (OMRON Corp.) |
| Liver | AST | Japan Society of Clinical Chemistry transferable method | Measurements were performed by LSI Medience Corp. |
|  | ALT | Japan Society of Clinical Chemistry transferable method | Measurements were performed by LSI Medience Corp. |
|  | GGT | Japan Society of Clinical Chemistry transferable method | Measurements were performed by LSI Medience Corp. |
| Inflammation | High Sensitive C-reactive Protein | Nephelometric method | Measurements were performed by LSI Medience Corp. |
|  | Interleukin-6 | Chemiluminescence enzyme immunoassay | Measurements were performed by LSI Medience Corp. |
|  | Tumor necrosis factor-alpha | Enzymatic method (CLEIA) | Measurements were performed by LSI Medience Corp. |
